# A strategy for trade monitoring and substitution of the organs of threatened animals

**DOI:** 10.1038/srep03108

**Published:** 2013-10-31

**Authors:** Jiao-yang Luo, Dan Yan, Jing-yuan Song, Da Zhang, Xiao-yan Xing, Yu-mei Han, Mei-hua Yang, Xiao-ping Dong, Cheng Peng, Shi-lin Chen, Xiao-he Xiao

**Affiliations:** 1 China Military Institute of Chinese Medicine, Integrative Medical Center of the 302 Military Hospital, Beijing 100039, P.R. China; 2 Institute of Medicinal Plant Development, Chinese Academy of Medical Sciences and Peking Union Medical College, Beijing 100094, P.R. China; 3 College of Pharmacy, Chengdu University of Chinese Traditional Medicine, Chengdu 610075, P.R. China; 4 These authors contributed equally to this work.

## Abstract

The use of threatened animals as a source of traditional medicines is accelerating the extinction of such species and imposes great challenges to animal conservation. In this study, we propose a feasible strategy for the conservation of threatened medicinal animals that combines trade monitoring and the search for substitutes. First, DNA barcoding provides a powerful technique for monitoring the trade of animal species, which helps in restricting the excessive use and illegal trade of such species. Second, pharmacological tests have been adopted to evaluate the biological equivalence of threatened and domestic animals; based on such testing, potential substitutes are recommended. Based on a review of threatened animal species and their substitutes, we find that the search for substitutes deserves special attention; however, this work is far from complete. These results may be of great value for the conservation of threatened animals and maintaining the heritage of traditional medicine.

Despite legislation from the Convention on International Trade in Endangered Species (CITES) and the establishment of the International Union for Conservation of Nature (IUCN) Red List^1^, threatened animal organs are still widely used in many countries, such as those of East and Southeast Asia^2^,^3^. The excessive use of animal organs as a source of traditional medicine (TM) is one of the main reasons why a number of species are threatened or endangered^4^. Some TMs are known to include trade-restricted species of animals as ingredients and therefore contravene the CITES legislation. For example, rhinoceros horn powder^5^, saiga antelope horn powder^6^ and bear bile crystals^7^ are commonly used in traditional Chinese medicines (TCMs) and occupy a special position in traditional Asian medicine; however, the biological origins of species have been decreasing rapidly in recent decades due to their increased use. Several international unions, such as the World Society for the Protection of Animals and the Wildlife Conservation Society, have attached great importance to these problems. Ascertaining the biological origin of these materials, enforcing legislation and prosecuting cases of illegal trade are important ways of conserving wildlife. To our knowledge, ingredients containing animal organs are presented mostly in the form of powders, tablets and capsules, and the biological origins of these materials are difficult to recognize using regular methods, such as chromatographic analysis, after they have been processed^8^. DNA barcoding, with its unique reproducibility, sequence versatility, and comparability among different species, offers a powerful approach to genetically authenticate animal organs^9^,^10^,^11^.

However, these traditional medicines have been the primary treatment for more than 3,000 years and have been accepted by a large portion of the population; therefore, they are difficult to eliminate^12^. Seeking substitutes that possess equipotent bioactivities from close phylogenetic species has been given much attention worldwide by ecologists, pharmacologists, economists and sociologists^13^,^14^; these efforts will greatly alleviate the pressure on threatened species. Liu *et al*. investigated substitutes by elucidating the active components on specific pharmacological activity, e.g., by purifying novel antioxidant peptides^15^. However, species authentication and comparative pharmacology of endangered animal-derived drugs (ADDs) and their substitutes should be considered to verify the reliability of possible substitutes.

This study aims to propose a strategy for wildlife conservation by combining trade monitoring and the search for substitutes (Fig. 1). Fifteen animal species were investigated, including three CITES-listed species (see the appendices at www.cites.org): saiga antelope (*Saiga tatarica*, listed in Appendix II), yellow-margined box turtle (*Cuora flavomarginata*, listed in Appendix II) and Chinese pangolin (*Manis pentadactyla*, listed in Appendix II); five state-protected species: black rhinoceros (*Diceros bicornis*, listed as critically endangered in the IUCN Red List), Père David's deer (*Elphurus davidianus*, listed as extinct in the wild in the IUCN Red List), sika deer (*Cervus nippon*, listed at the first state protection level in China), red deer (*Cervus elaphus*, listed at the second state protection level in China), Chinese pond turtle (*Chinemys reevesii*, listed as endangered in the IUCN Red List); and seven domestic species: sheep (*Ovis aries*), domestic goat (*Capra hircus*), Asian water buffalo (*Bubalus bubalis*), Chinese softshell turtle (*Trionyx sinensis*), hawksbill (*Eretmochelys imbricate*), yellow-headed tortoise (*Indotestudo elongata*), and yellow-bellied slider turtle (*Trachemys scripta*). The samples genetically audited in this study using DNA barcoding are shown in Fig. 2. DNA barcoding was applied to authenticate the animal species, which involved the DNA sequencing and characterization of mitochondrial DNA (mtDNA) genes. This technique is able to identify threatened species and domestic species with confidence, thereby offering a reliable tool for customs officials. In addition to concerns about trade monitoring, the lack of substitutes has also affected threatened animal species. For this reason, we adopted various pharmacological methods to evaluate the bioactivities of several ADDs using their biological fingerprints and characteristics. We expected to find equivalence regarding the specific functions of ADDs obtained from threatened species to derive substitutes for use in complementary and alternative medicines. The design of the present study is depicted in Fig. 1.

## Results

### Sequence information, polymerase chain reaction amplification capacity, and sequencing success rate

Polymerase chain reaction (PCR) amplification success and sequencing success are important indices for evaluating DNA barcodes. In the present study, both PCR amplification and sequencing achieved a 100% success rate. The PCR amplification electropherogram was obtained using agarose gel electrophoresis. Using DnaSP version 5.10.01, 72 sequences (Fig. 3) were analyzed, and 477 sites (i.e., base pairs) were obtained; 463 sites were without gaps or missing data. Of these, 156 sites (32.7%) were polymorphic; the nucleotide diversity (pi) was 10.9%. The average nucleotide composition of all the sequences was calculated in MEGA 4.0^16^ as follows: T = 31.3%; C = 24.8%; A = 28.5%; and G = 15.4%; the average AT content was 59.8%.

### Intra- and interspecific genetic divergence analysis

For successful DNA barcoding, the sequence variation between species needs to be sufficiently high so that they can be distinguished from one another; however, the variation must be sufficiently low within species that a clear threshold between intra- and interspecific genetic variations can be defined. In the present study, the mean intra- and interspecific genetic variations were 0.4% and 15.8%, respectively. All intraspecific variations measured between sequences were less than 1.8%, and all interspecific genetic variations were greater than 2.0%.

### Phylogenetic analyses

A cladogram was clustered based on neighbor-joining analysis and demonstrated that all ADD haplotypes were strongly grouped with their orthologous mtDNA. We also noted that with eight species, the ADDs were clustered in four subfamilies in the dendrogram (Fig. S2).

To test the accuracy of the barcodes and expand our identification capability, we downloaded all complete mitochondrial cytochrome *c* oxidase I (COI) genes available in GenBank (November 2011). All 241 sequences (Table S1) were cropped to the 477-base pair barcode region amplified by the primer pair MahF1 and MahR1. A phylogenetic analysis of 313 sequences was conducted, which included the GenBank dataset and our test sample dataset (Fig. 3). The cluster branches indicated that all haplotypes strongly grouped with their orthologous mtDNA and every species clustered in the appropriate branch. Based on this analysis, we were able to deduce that this region of the COI gene is a good candidate for distinguishing between the ADDs and between the taxonomy of related species.

### Barcoding gap test and identification capability

Ideal DNA barcodes should contain a conspicuous spacer region that delineates intra- and interspecific variations — the barcoding gap^17^,^18^. In the present study, a visible barcoding gap was found in only a small portion of the ADDs with overlapping intra- and interspecific variation (Fig. S3); this indicated that all ADDs could be accurately identified.

Comparing species-identification capability relative to other genetic fragments is one of the criteria for evaluating the quality of various barcodes. In this study, the taxonomic affiliation of each sequence (including those of 72 test samples and 241 individuals from GenBank) was determined using BLAST1 against the GenBank database. This analysis indicated that all mitochondrial sequences were successfully identified.

### *In vivo* study of endotoxin-induced rabbit fever

The effects of the ADDs on the rabbit fever models are shown in Fig. S4A and Table S2. As is evident from Fig. S4A, the blank control had a wave range of 0.1°C, which indicates a successful fever model. Compared with negative controls, saiga antelope horn, pangolin scale, goat horn, and aspirin significantly reduced the temperature of fevered rabbits; velvet antler and antler significantly enhanced the temperature of fevered rabbits. Saiga antelope horn exhibited the strongest anti-fever effect, followed by pangolin scale and goat horn.

The endotoxin, PGE1, PGE2, and TNF-α levels in the serum of rabbits were determined to establish the effective mechanisms of the ADDs. Compared with negative controls, saiga antelope horn and goat horn clearly reduced the levels of PGE1, PGE2, and TNF-α, although velvet antler caused significant increases (Figs. S4B–S4D). Blood endotoxin content was higher when using velvet antler compared with the other ADDs (see Table S2). It has been established that endotoxins stimulate monocytes and macrophages to produce proinflammatory cytokines, including TNF-α and IL-1β^19^,^20^, which subsequently cause fever. We demonstrated that saiga antelope horn and goat horn inhibited the production of TNF-α and PGE2, thereby relieving fever.

### *In vitro* study of liver microsomes

Thermogenic curves of the growth of liver microsomes recorded at 37°C under the action of ADDs are shown in Fig. S5A. The heat-flow power (HFP) time curve could be delineated using the following equation^21^: *P_t_* = *P*_0_ exp(*kt*) or ln*P*_t_ = ln *P*_0_ + *kt*, where *P*_0_ and *P*_t_ represent the HFP at time 0 and t (min), respectively. From this equation, the growth-rate constants (*k*) of the exponential phase for microsomal metabolism at 37°C in the absence of any substance could be calculated using the data obtained for the highest peak. Then, quantitative thermokinetic parameters, such as the HFP of the highest peak (*P*), the appearance time of the highest peak (*t*), and the total heat output (*Q*_t_) were obtained from the HFP time curve of the microsomal growths affected by the various ADDs (Fig. S5B).

The inhibition ratio (*I*) of ADDs on microsomal growth was calculated from *k*. Using principal component analysis^22^, *P* and *Q*_t_ were calculated as the main components, accounting for 91.89% of the variability. The inhibition ratio was calculated using the following equation: *I* = (*P*^t^ − *P*^0^)/*k*^0^, where *P*^0^ and *P*^t^ represent the HFP in the absence of any substance and in the presence of the ADDs, respectively. By analyzing the main components, we noted two findings. Saiga antelope horn and goat horn inhibited the heat output power and the total heat output, which inhibited microsomal growth. In addition, velvet antler and antler enhanced the heat output power and the total heat output, which indicated increased heat output by the microsomes.

### *In vitro* study of spleen lymphocyte proliferation

The thermogenic curves at 37°C in the presence of ADDs are shown in Fig. S6E. Based on the liver microsomal study, *k*, *P*, *t*, and *Q*_t_ were obtained; among these, *P* and *Q*_t_ were identified as the main components. From an analysis of these main components, we determined that velvet antler and antler increased *P* and *Q*_t_ to varying degrees, and saiga antelope horn, pangolin scale, goat horn, and buffalo horn clearly inhibited the heat output of the spleen lymphocytes (Figs. S6F, S6G). These results agree with the theory of the “nature of heat and cold” in traditional Chinese medicine^23^. Because lymphocytes are closely related to immunization^24^,^25^, we believe that ADDs differ in their immunomodulatory effects. To a certain extent, we also consider that ADDs from the horns of threatened animal species can be partially substituted by other substances.

### *In vivo* study of thermotaxis in mice

The nature of heat and cold is an intrinsic property of matter^23^. In this part of the study, animal behavior was investigated to explain the nature of ADDs. Mice were randomly divided into 6 treatments, consisting of 10 replicates. Investigations were carried out under the following temperature gradients: 15–30°C, 15–40°C, 20–30°C, 20–40°C, 25–40°C, and 30–40°C. Under these conditions, the mice exhibited an obvious temperature tropism: they congregated in either the warm zone or the cool zone. At 15–25°C, 15–35°C, 20–35°C, or 25–35°C, the mice showed no obvious temperature tropism: the numbers of animals in the warm and cool zones were similar (Fig. S7). For mice not exposed to ADDs, equal numbers were found in the two zones for the first five hours. However, beyond that time, the numbers of animals in the zones changed significantly, as did the 30-min movement distances and the number of times the animals crossed from one zone to another (Fig. S8). Thus, the results were repeatable within the first five hours.

The results of thermotaxis in mice exposed to ADDs showed that velvet antler and antler reduced the number of animals in the warm zone and increased the 30-min movement distance and the number of times the mice crossed from one zone to another (Figs. S9D – S9F). In contrast, saiga antelope horn, goat horn, and pangolin scale promoted the movement toward heat. There was no significant difference in the effects of saiga antelope horn, goat horn, and pangolin scale on thermotaxis (Figs. S9D – S9F). In addition, velvet antler and antler increased the autonomic action of the mice, whereas saiga antelope horn, goat horn, and pangolin scale decreased this action.

In addition to studying animal behavior, we determined the levels of 5-hydroxytryptamine (5-HT), cyclic adenosine monophosphate (cAMP), and cyclic guanosine monophosphate (cGMP) in the serum and brain tissues of the mice. 5-HT is an inhibitory neurotransmitter in the vertebrate nervous system that plays an important role in controlling mental states, perception, and muscular fatigue. We found that saiga antelope and pangolin scale significantly enhanced the level of 5-HT in both the brain tissues and sera of mice compared with blank controls. However, velvet antler, antler, and goat horn showed no significant effect on 5-HT levels (Fig. S9G; Fig. S10). cAMP and cGMP are cyclic nucleotides that exert antagonist effects *in vivo*^26^. The balance between cAMP and cGMP is essential for cellular metabolism and plays a role in many pathological processes^27^. We determined that velvet antler and antler significantly enhanced the level of cAMP in both the brain tissues and sera of mice compared with blank controls (Fig. S9H); velvet antler also clearly enhanced the level of cGMP in both the brain tissues and sera of mice (Fig. S9I). Conversely, saiga antelope horn, goat horn, and pangolin scale decreased cAMP and cGMP levels to various degrees (Figs. S9H and S9I). Moreover, saiga antelope horn significantly increased cAMP/cGMP values, whereas velvet antler decreased these levels (Fig. S9J).

### *In vitro* study of thrombus formation^28^

We evaluated the rabbit plasma recalcification and thrombin times of ADDs; saiga antelope horn, pangolin scale, yak horn, and buffalo horn significantly increased the rabbit plasma recalcification (*p* < 0.01) and thrombin (*p* < 0.01) times; in addition, these drugs exhibited good anticoagulant effects. Antler, goat horn, and turtle shell also increased the rabbit plasma recalcification (*p* < 0.05) and thrombin (*p* < 0.05) times. Saiga antelope horn and Mongolian gazelle horn had no obvious effect on thrombus formation (*p* > 0.05).

## Discussion

Threatened animal conservation is a special field within applied ecology. Currently, the objectives of wildlife management fall into the following categories: protecting the natural habitats of animals through controlled exploitation; maintaining protected areas containing rare species; establishing biosphere reserves for endangered species; protecting wildlife through legislation; and imposing specific restrictions on the export of endangered animals or their products. However, it is undeniable that many drugs derived from threatened animals have remarkable curative effects in traditional medicine. We consider that the most effective way of protecting medicinal wild animals is to combine trade monitoring and seeking substitutes.

Regarding trade monitoring, the techniques applied include macroscopic and microscopic morphology, histology and molecular biology; however, these methods fail to identify ADDs with confidence. In recent decades, molecular techniques have promoted the identification of ADDs; these techniques include protein electrophoresis, restriction fragment length polymorphism analysis, and using specific primers such as those for 12S rDNA, 16S rDNA, valine tRNA and Cyt B to amplify specific regions in the mitochondrial genome. However, these markers have no universal primers for broad animal species. Despite the great potential of genetics to assist in the identification of ADDs and the phylogeny of some animal clades, such as the Bovidae, Cervidae, and Moschidae^29^,^30^,^31^, little consensus exists regarding which gene region is the most suitable for studying a wide range of animal species. Recently, the mitochondrial cytochrome c oxidase I (COI) gene has attracted global attention as a DNA barcode for animals because the consortium for the barcode of life (CBOL) uses universal primers to amplify a region of the COI gene approximately 650 bp in length to identify broad animal taxonomy. Miller^32^ considered that the DNA barcode technique might usher taxonomic research into a new era. The COI gene is sequenced to yield a DNA barcode for the examined specimen for use in trade monitoring; this barcode is compared to barcodes from reference specimens to identify the species. Our results indicate that the COI gene is a useful barcode for the trade monitoring of threatened animal species, in agreement with a previous report^33^ demonstrating that even short COI fragments can efficiently identify antelopes, buffalo and domestic Bovidae; thus, this technique exhibits great potential for use in wildlife conservation. DNA barcoding can serve as a powerful tool in wildlife forensics^34^,^35^ and might prove to be a vital aid in conserving organisms that are threatened by the illegal wildlife trade, such as turtles^36^. The COI gene has also enhanced our understanding of mammalian diversity in Southeast Asia and, in this way, aided conservation planning^37^. The results obtained here confirm that the COI gene exhibits good universality and a powerful ability to identify animal species; therefore, this gene should play an important role in monitoring the trade in endangered animals. However, the heavy reliance on one gene for identification has its shortcomings for wildlife forensic enquiries, so that we suggest that if COI gene could not be obtained, other protein coding gene may be a good backup.

Animal products play an important role in traditional medicine within China^38^, Japan^39^, Brazil^40^ and many other countries. Although some animal species are becoming threatened or endangered due to their excessive use as complementary medicine, few measures have been proposed for the sustainable development of traditional medicine. Emphasizing conservation biology while disregarding the traditional use of wildlife in medicine will therefore encounter great resistance. One feasible solution is to substitute the organs of closely related domestic animals by examining their biological properties and ranking the most relevant indices regarding their medicinal values. In the present study, several pharmacological tests were performed on ADDs obtained from both threatened and domestic animals, and the resulting bioactivities (both *in vitro* and *in vivo*) were compared in several ways.

Fig. 4 presents several regularities. 1) Velvet antler and antler show opposite tendency compared with other ADDs, e.g., saiga antelope, domestic goat, Chinese pangolin and Chinese softshell turtle reduce the temperature of fevered rabbits while velvet antler and antler enhance that. This regularity is validated by the tests of liver microsome, spleen lymphocytes and thermotaxis. 2) The levels of the bioactivities among ADDs vary in different tests. For example, saiga antelope and domestic goat share the same level of activity on temperature and thermotaxis tests; while the activity of saiga antelope is better than that of domestic goat on liver microsome (*p* < 0.05) and spleen lymphocytes tests (*p* < 0.05). Another example is that velvet antler and antler share the same level of activity on spleen lymphocytes test while different regarding other tests. 3) Two new bioactivities are found including the antipyretic activity of Chinese pangolin and the anticoagulant activity of velvet antler. 4) ADDs derived from threatened species that may be replaced by ADDs obtained from domestic animals (designated by different colors in the figure) would otherwise be difficult to substitute. We conclude that domestic goat may be the potential substitute of saiga antelope regarding its immunological and antipyretic activities, and Chinese softshell turtle may be the potential substitute of Chinese pangolin regarding its immunological activity. These potential substitutes could alleviate the burden on threatened species and enrich the traditional medicines. 5) Although the bioactivities of antler are not so good as velvet antler, the increase dosage of the antler may achieve the same potency. This substitutive use can reduce harm to deer since antler is ossific angle that will shed naturally once a year while velvet antler is obtained by sawing.

Pharmacological studies indicate that each ADD usually has several therapeutic benefits and that only certain actions of threatened-ADDs are interchangeable. Based on the results of this study, we are able to establish a criterion for the use of ADD substitutes: if a substitute shows the same level of bioactivity as that of a threatened ADD for specific functions, it may be adopted as a candidate for potential clinical use. We believe that further study in this area along the suggested lines would contribute to the use of substitutes. However, it should be noted that the replacement of one particular function does not equate to complete replacement. Thus, when examining possible candidates for substitution, all of the main functions or bioactivities of the ADDs need to be examined and clinical trials should be conducted where appropriate^41^. Additionally, the mechanisms of the ADD bioactivities have to be clarified to aid in identifying possible substitutes^42^,^43^: ADDs derived from domestic species that share the same mechanisms as those obtained from threatened species should be given the highest priority.

But *et al*. pointed out an important aspect for the remedies of endangered animal species by using medicinal plants, which are easier to obtain^5^. If so, it should be good news for conservation of wild animals. However, to our knowledge, medicinal plants were only adopted by few cases to replace animal-based remedies. Feng *et al*. reported that plant substitutes were only suggested in literature without solid evidence to support, while some animal substitutes were superficially studied^44^. Even if efforts on finding the alternative domestic animals for the endangered animals are also not many, we added Table S4 to summarize current investigation. In addition, when it is not possible to replace the medicinal value of ADDs from threatened species with those from domestic species, efforts should be made to examine the relevant active components in the ADDs and investigate any interactions among the various constituents; this would be useful in the attempt to synthesize alternatives. Otherwise, it would be necessary to direct funding toward those species that are at the greatest risk of extinction. However, this strategy is not the most efficient way to promote the recovery of such species or reduce global extinction rates; some of the species at the greatest risk of extinction would require a massive recovery effort, most likely with a small chance of success^45^.

We have summarized the trade monitoring and substitution of threatened animal species (Table S4), which has attracted the strongest concern worldwide; species at risk include cetaceans, carnivora, proboscidea, pholidota, perissodactyla, artiodactyla, the reptiles testudines and the fish pleurotremata. The threatened species are listed in order according to their position on the IUCN Red List of threatened species. Among the listed species, 42 are included in the CITES appendices, which afford different levels of protection from successive use^46^. Table S4 demonstrates that studies of the substitution of threatened animal species have just begun, and many critically endangered species, such as Père David's deer, saiga antelope and hawksbill turtle, cannot be substituted and have no alternative uses. Many articles have been written about the use of threatened animal species in traditional medicines^47^,^48^,^49^,^50^,^51^, and the illegal trade in these species for medicinal use will possibly destroy biodiversity^52^,^53^,^54^. Therefore, the development of substitutes for drugs derived from threatened species, critically endangered species in particular, is of great significance for wildlife conservation.

The use of substitutes not only relates to the bioactivities of these drugs, it also involves traditional and cultural concerns. The functions of organs threatened animals are often exaggerated based on value orientations, religious beliefs, superstitions and totem culture, and the blind faith of some high-end consumers, with complete disregard for the exploitation of wild animal resources.

## Methods

### Taxon sampling

**Figure 2:**
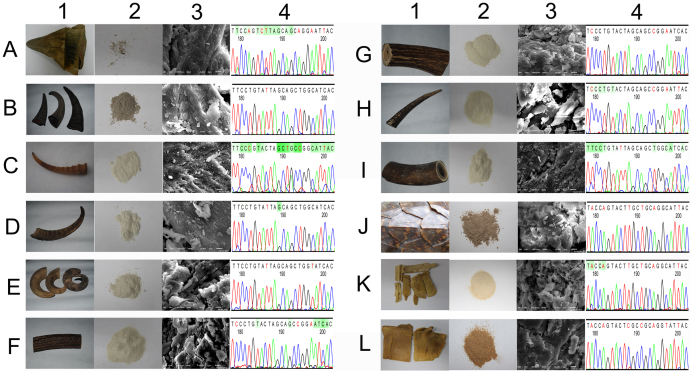
Sample information. (A): black rhinoceros; (B): Asian water buffalo; (C): Mongolian gazelle; (D): domestic goat; (E): sheep; (F): red deer; (G): sika deer; (H): Père David's deer; (I): Chinese muntjak; (J): Hawksbill; (K): Chinese softshell turtle; (L): Reeve's turtle. 1: Photograph of animal organs; 2: powdered animal organs, which represent the main market form and which are hard to discriminate; 3: a scanning electron microscope photograph of animal organs (obtained using a JSM-6510 instrument, Japan; gold sputtering treatment: 40 mA, 130 s; SEI: 12 kV; WD: 12 mm; amplification × 5500), no typical diagnostic characteristics are found; 4: sequencing peaks (the peaks were clipped using Codoncode Aligner, and the 24-bp segment can be used to identify all of the animal organ samples). (The photographs in this figure were taken by JL, and we hold the copyright of this figure).

**Figure 3:**
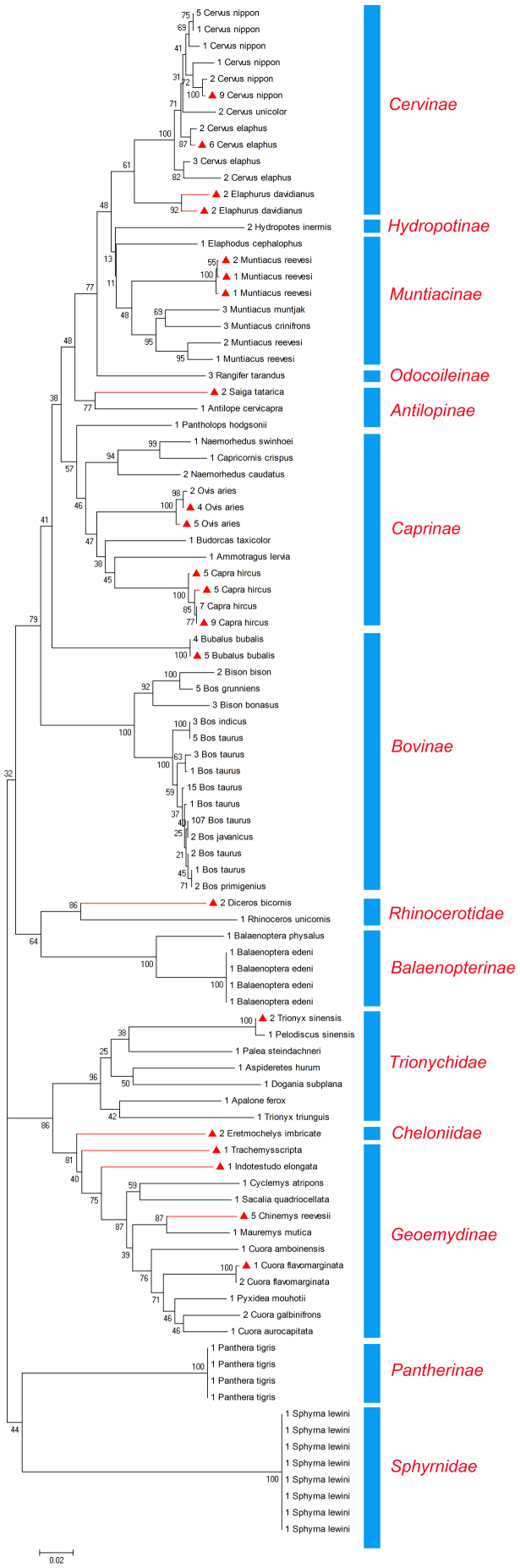
Neighbor-joining tree of 313 COI complete gene sequences available at GenBank. The red triangle represents the experimental individuals; the numbers in front of the taxon names are the species identification numbers.

**Figure 4:**
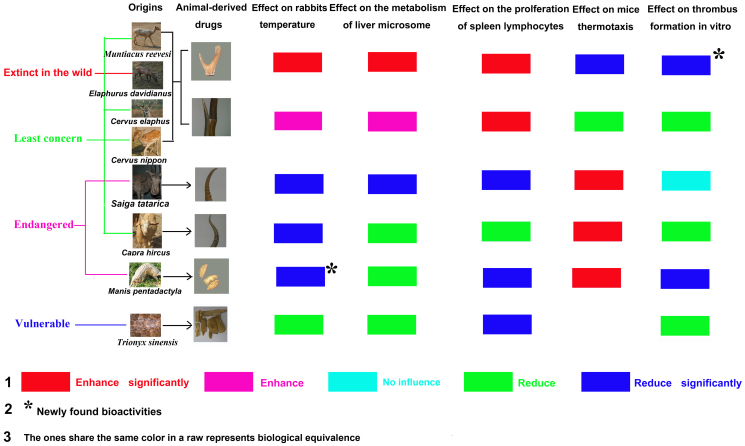
Comprehensive analysis and conclusions of this study. The activity of each sample is designated by different colors in the figure, and the samples that share the same color in a raw are biologically equivalent. (The photographs in this figure were taken by JL and DY, and we hold the copyright of this figure).

A total of 72 animals from 15 species that are used as sources of ADDs were collected in six provinces and regions of China (Beijing, Jilin, Sichuan, Qinghai, Xizang, and Hubei). The sample information is depicted in Fig. 2. Among the samples, those derived from wild animals, including saiga antelope horn, *Cervus nippon* velvet antler and *Cervus nippon* antler, were provided by the Animal Research Institute of the Chinese Academy of Sciences and China's Forestry Sciences (Table 1). The distribution information was derived from investments made in TM in China (Fig. S1). We also analyzed 241 animal sequences within four related families from GenBank. We received a license from the State Forestry Administration of China to study wild animals, and the license number is Lam Woo Approval [2005] 627; this license is listed on the official website of the State Forestry Administration of China (http://www.forestry.gov.cn). Throughout this study, none of the authors handled any animals. Although we investigated animal products, we had no direct contact with the animals themselves. The samples were collected by administrators in the National Nature Reserve and at aquaculture bases.

### DNA extraction

ADDs was frozen at −4°C and subsequently ground in a DNA extraction beveller (Retsch MM400, Germany) for 1 min at 1,800 rpm. The total DNA was then extracted using DNAout kits. Specifically, 200 mg of each ADD sample was dispersed in 1 ml DNAout solution, and 10 μl RNase A and a little silicon were subsequently added. After incubating for 5 minutes at 65°C, the solution was centrifuged at 15000 × *g* for 5 minutes, and the supernatant was extracted twice with chloroform, each time 0.2 ml. Then, an equal volume of isopropyl alcohol was added into the supernatant, shaken up, and centrifuged at 15000 × *g* for 5 minutes. The precipitation was washed twice with ethanol, each time 1 ml, and then dissolved in buffer solution for use. The PCR primers were designed from a ClustalW alignment of COI sequences available from GenBank for 54 closely related species within the Bovidae, Cervidae, Geoemydidae and Trionychoidae families. The forward primer (MahF1 5'-GCAGGAACAGGCTGAACCGT-3') was anchored at site 364 (the numbering used is relative to the complete CO1 sequence from the above four families); the reverse primer (MahR1 5'-AATATGTGGTGGGCTCATAC-3') was anchored at site 863. The performance of MahF1 and MahR1 in PCR amplification was tested using genomic DNA isolated from 10 Bovidae, Cervidae, Geoemydidae, and Trionychoidae species. Preliminary PCR amplifications using MahF1 and MahR1 achieved a 100% success rate.

### PCR amplification

PCRs were performed in 25 μl volumes containing 13.3 μl of PCR-grade water, 2.5 μl of 10 × PCR buffer, 2.0 μl of magnesium chloride (2.5 mM), 1.0 μl of each primer (2.5 μM), 2.0 μl of deoxynucleotide triphosphates (2.5 mM), 0.2 μl of Taq polymerase (5 units μl^−1^), and 1.0 μl of DNA extract. The cycling conditions were as follows: an initial step of 3 min at 94°C followed by 33 cycles of 30 s at 94°C, 30 s at 58°C, and 60 s at 72°C, followed by 10 min at 72°C. The primers MahF1 and MahR1 were used to amplify a 477-base pair fragment of COI for all samples. The PCR products were electrophoresed in 1.0% tris-borate-EDTA agarose gels, stained with ethidium bromide, and visualized under ultraviolet light.

### DNA sequencing

The PCR products were directly sequenced with the same primers in 20 μl reactions containing 2.0 μl of 5 × sequencing buffer, 1.0 μl of each primer (2.5 μM), 13.3 μl of PCR-grade water, 0.2 μl of BigDye (Applied Biosystems, USA), and 1.0 μl of PCR product. The cycling conditions were as follows: an initial step of 2 min at 95°C, followed by 30 cycles of 15 s at 96°C, 15 s at 52°C, and 4 min at 60°C. Sequencing reactions were performed in both directions using the PCR primers. Sequencing products were purified with Sephadex G-50 (Sigma-Aldrich Co., USA) columns in Millipore multiscreen HV filter plates and then analyzed using an ABI 3730XL DNA analyzer (Applied Biosystems, USA). The resulting sequences were assembled, edited, and aligned in Seq-Scape V.3.0 (Applied Biosystems, USA) before being uploaded to the Barcode of Life Data System.

### DNA analyses

Phylogenetic analyses were performed on 313 mitochondrial COI sequences, including 72 newly generated mitochondrial COI sequences (followed by GenBank Accession Numbers) and 241 pre-existing sequences downloaded from GenBank. Haplotype distribution and the number of polymorphic sites were estimated using the software DnaSP 5.10.01^55^. All taxa were subjected to pairwise sequence divergence calculations using the Kimura-2-parameter (K2P) model in MEGA 4.0 software^16^ because this model can provide the best metric when genetic distances are low^56^. A neighbor-joining (NJ) tree with bootstrap analysis was constructed using MEGA 4.0. Based on the COI variants, species identification was carried out using BLAST1 as described previously by Ross et al.^57^.

### Ethics statement for the pharmacodynamic study

This study was conducted in strict accordance with the recommendations of the Guidelines for the Care and Use of Laboratory Animals of the Ministry of Science and Technology of China. The animal protocol was approved by the Committee on the Ethics of Animal Experiments of the 302 Military Hospital.

### Animals

Kunming mice, male, weighing 20 ± 2 g, were obtained from the Laboratory Animal Center of the Academy of Military Medical Sciences (License No. SYXK 2007-004) and adopted for the experiments of liver microsomes, spleen lymphocyte and thermotaxis. New Zealand pure big-ear white rabbits, weighing 1.5 ± 0.2 kg, were obtained from the Laboratory Animal Center of the Academy of Military Medical Sciences (License No. SCXK [Beijing] 2007-0003). The animals were raised separately by gender and had unlimited access to food and water in an environmentally controlled breeding room (temperature 22 ± 2°C, humidity 60 – 80%). The breeding room was illuminated by an artificial light cycle with 12 hours of light and 12 hours of darkness every day and was disinfected regularly.

### Sample preparation for the pharmacological study

Because of the limitation of the amount of some ADDs samples, only six ADDs derived from a total of eight species were tested for pharmacological studies. Each ADD was weighed (approximately 300 g) and immersed in cold water for 1 hour. Then, 1,500 ml of water was added; this was decocted three times, once an hour. Next, the decoction solutions were combined and concentrated to 1,000 ml to achieve a final solution equivalent to 0.3 g ingredient ml^−1^. The concentrate was then sealed and sterilized using high-pressure steam at 121°C for 30 min and stored at 4°C. In the pharmacological study, all ADDs underwent the same preparation process, and various concentrations were obtained by diluting the mother liquid with distilled water.

### *In vivo* study of endotoxin-induced rabbit fever

Healthy New Zealand pure big-ear white rabbits were subjected to frequent temperature measurements. Animals were selected whose temperatures were 38.6–39.5°C and did not vary more than 0.3°C. The selected rabbits were randomly allocated into nine groups with six rabbits in each group (including six experimental groups, a positive control and blank control). *Escherichia coli* endotoxin (O111B4) was slowly injected into the ear vein of rabbits with the concentration of 12 EU/kg to build fevered rabbits models. After one hour, ADDs (5.0 ml mother liquid kg^−1^) were administered to rabbits and the change in the rabbits' body temperature was measured to monitor the effects of ADDs. Positive controls were administered 200 mg kg^−1^ aspirin, and negative controls were administered the same volume of water and endotoxin in the ADD groups; blank controls were given only the same volume of water in the ADD groups. ADDs were administered to rabbits 1 hour before fever was induced, and the initial temperatures were immediately measured. The body temperature of the rabbits was measured at 0.5, 1.0, 1.5, 2.0, 3.0, 4.0, 5.0, and 6.0 h after fever was induced. Ear-vein blood samples were taken from the rabbits, and PGE1, PGE2, and TNF-α levels were measured by radioimmunology. The endotoxin content was determined using a dynamic nephelometric method. In pre-interference experiments, the same amount of endotoxin was added, and the recovery rates were calculated (Table S2).

### *In vitro* study on spleen lymphocyte proliferation

Ten healthy Kunming mice were weighed and immediately sacrificed under anesthesia. The spleens were obtained, and the residual blood and polyp tissues attached to the spleen were washed away using phosphate-buffered saline; dispersed cells were prepared by passing the tissue through a cell sieve (mesh size, 75 μm). The cell suspension was then centrifuged for 5 min at 1,000 × g, and the pellet was suspended in Dulbecco's modified Eagle's medium culture to a density of 5 × 10^6^ cell ml^−1^. Then, 100 μl of the cell suspension was added to each well of 96-well culture plates, and 100 μl aliquots of Dulbecco's modified Eagle's medium containing various doses of the ADDs were added to each well. Finally, the 96-well culture plates were incubated for 72 h at 37°C in 5% CO_2_, and the cytopathogenic effect was microscopically observed.

Preliminary study showed that ADDs did not cause damage to spleen lymphocytes at concentrations lower than 40 mg ml^−1^ (Table S3), and 10 mg ml^−1^ was finally selected for the present experiment. Microcalorimetry^58^,^59^ was used to determine the HFP curves for spleen lymphocyte proliferation. To achieve a cell density of 5 × 10^4^ cell ml^−1^, 5 ml of RPMI-1640 culture incubated in mouse spleen cells was added quantitatively to a 20-ml ampoule under sterile conditions. Then, 500 μl of RPMI-1640 culture containing one type of ADD (10 mg ml^−1^) was added to the ampoule. Ampoules containing various ADD and spleen cell suspensions were sealed and lowered into the measuring position of the calorimeter block. After approximately 30 min (by which time the temperature of the ampoules had reached 37°C), HFP time curves were recorded until the readings had returned to the baseline level. All data were continuously recorded using a dedicated software package (PicoLog TC-80, TA Corporation, USA).

### *In vitro* study using liver microsomes

The liver tissues of the ten killed mice in above test were taken and placed into iced water. The fat tissue were quickly removed and discarded; a sufficient amount of the tissue was then homogenized with approximately 10 strokes using a glass Dounce homogenizer. A volume 10 times that of the mitochondrial extract (including 0.4 M mannitol, 50 mM tris-hydroxymethyl aminomethane-2 hydrochloric acid, 1 mM disodium ethylenediamine tetraacetate, 5 mM potassium chloride, 0.1% bovine serum albumin and 2 mM mercaptoethanol at pH 7.5) was required to ensure extraction efficiency. The homogenate was transferred to aseptic centrifuge tubes and centrifuged at 1,000 × *g* at 4°C for 5 min. The supernatants were transferred to new centrifuge tubes and centrifuged at 12,000 × *g* at 4°C for 10 minutes; the pellets were then collected and dissolved with phosphate-buffered saline for use as the mitochondrial suspension. The subsequent tests were determined using microcalorimetry, and the experimental protocol, groups and dosage were the same as the test of spleen lymphocyte.

### *In vivo* study on thermotaxis in mice

Kunming mice were fed and monitored for 3 days before the experiment, and those with no obvious temperature change were used for further training so that they could memorize the various experimental zones. Afterwards, the mice were randomly allocated into seven groups, with ten mice in each group. The experimental groups were administered one type of ADD for 7 days at a dose of 5.0 ml of mother liquid kg^−1^ day^−1^ before recording behavioral observations; the negative control was given the same volume of water. After the traces had been recorded, the mice were sacrificed, and blood samples were obtained after enucleating the eyeballs. Immediately thereafter, brain tissue was extracted.

The samples of blood were centrifuged for 5 min at 1,000 × g, and the resulting serum was stored at –20°C. The level of 5-HT in the blood was directly determined using a 5-HT ELISA Kit (Cusabio Biotech Co., USA). A mixture of 0.1 ml of serum with 2.0 ml of ethanol was evenly shaken and incubated at room temperature for 5 min before being centrifuged for 10 min at 1,500 × *g*. The supernatant was transferred to a new centrifuge tube, and the pellet was added to 1 ml 75% ethanol, evenly shaken, and centrifuged for 10 min at 1,500 × *g*; this second supernatant was collected and mixed with the first supernatant. The supernatant was dried at 60°C and dissolved with 1 ml of acetate buffer, which was then analyzed by radioimmunoassay.

Two milliliters of ethanol was added to 2 ml of brain homogenate (containing 50 mg of brain tissue), mixed well, incubated for 5 min, and centrifuged for 15 min at 1,500 × *g*. The supernatant was collected, dried at 60°C, and stored at 4°C. Half of the residue was dissolved in acetate buffer, and the cAMP and cGMP levels were determined by radioimmunoassay. The remaining half was dissolved in normal saline, evenly shaken, centrifuged for 10 min at 1,000 × *g*, and the 5-HT level was determined.

## Author Contributions

J.L. carried out the experiments, participated in the sequence alignment, and drafted the manuscript. D.Z. and Y.H. participated in the sequence alignment. D.Y., J.S., X.D. and M.Y. participated in the design of the study and performed the statistical analysis. X.X.H. and C.P. participated in the *in vitro* and *in vivo* experiments. X.X., D.P. and S.C. conceived the study, participated in its design and coordination, and helped to draft the manuscript. All authors have read and approved the final manuscript.

## Supplementary Material

Supplementary Information Supplementary Information

## Figures and Tables

**Figure 1:**
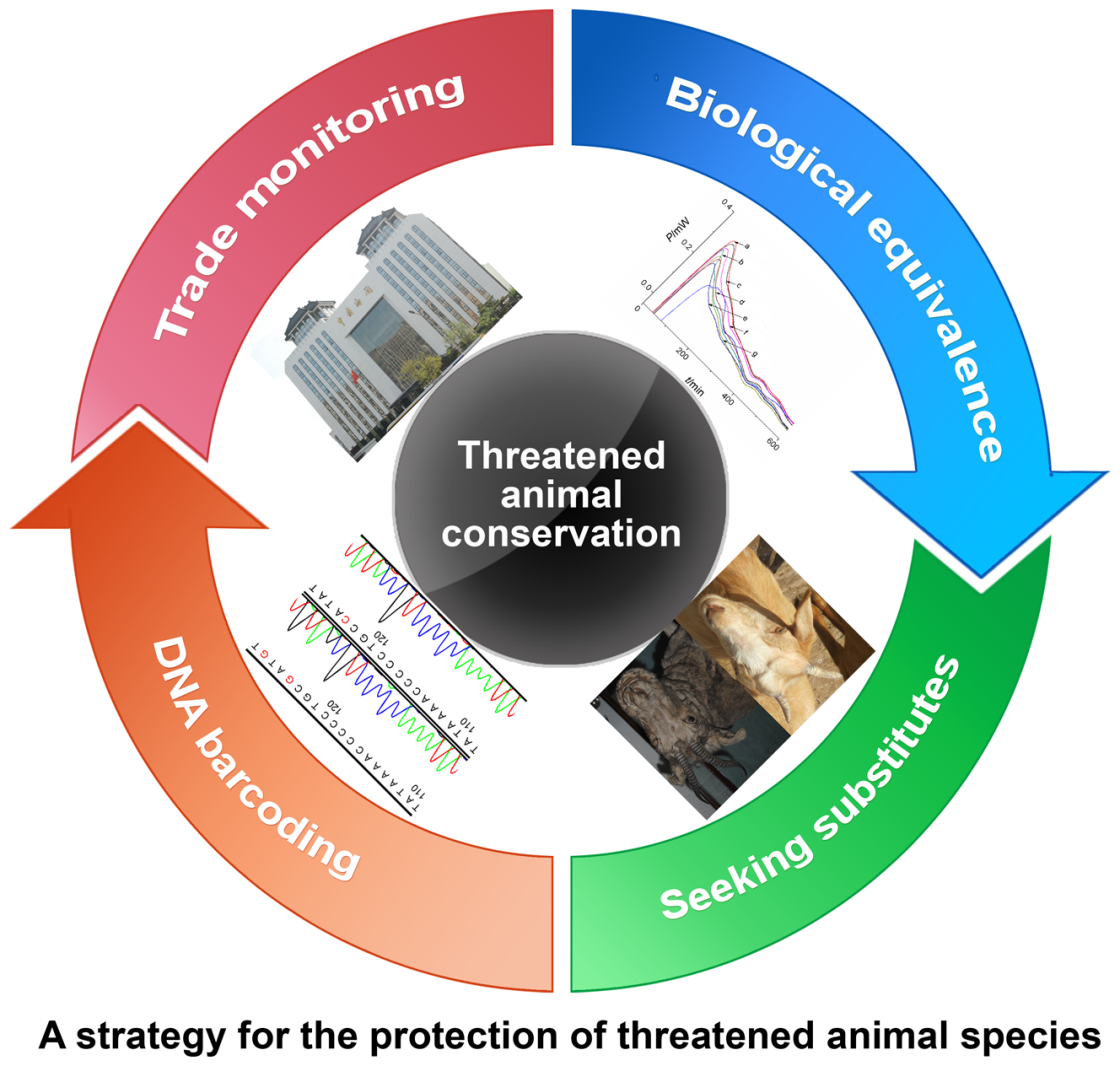
Study design. (The photographs in this figure were taken by JL and JS, and we hold the copyright of this figure).

**Table 1:** The studied species, including scientific names, common names, and status in IUCN and CITES legislation

Scientific name	Common name	Medicinal part	CITES legislation	IUCN Red List	Population trend
*Cervus elaphus*	Red deer	Velvet/antler	NL	Least concern	Increasing
*Cervus nippon*	Sika deer	Velvet/antler	NL	Least concern	Increasing
*Elaphurus davidianus*	Père David's deer	Velvet/antler	NL	Extinct in the wild	Increasing
*Muntiacus reevesi*	Chinese muntjak	Velvet/antler	NL	Least concern	Decreasing
*Saiga tatarica*	Saiga antelope	Horn	Appendix II	Critically endangered	Decreasing
*Eretmochelys imbricate*	Hawksbill	Shell	NL	Data deficient	Unknown
*Cuora flavomarginata*	Yellow-margined box turtle	Shell	Appendix II	Endangered	Needs updating
*Indotestudo elongata*	Yellow-headed tortoise	Shell	NL	Endangered	Needs updating
*Trachemys scripta*	Yellow-bellied slider turtle	Shell	NL	Least concern	Stable
*Bubalus bubalis*	Asian water buffalo	Horn	NL	Least concern	Unknown
*Capra hircus*	Domestic goat	Horn	NL	Least concern	Unknown
*Chinemys reevesii*	Reeve's turtle	Scale	NL	Least concern	Unknown
*Diceros bicornis*	Black rhinoceros	Horn	NL	Critically endangered	Increasing
*Trionyx sinensis*	Chinese softshell turtle	Scale	NL	Vulnerable	Decreasing
*Ovis aries*	Sheep	Horn	NL	Data deficient	Unknown
*Manis pentadactyla*	Chinese pangolin	Shell	Appendix II	Endangered	Decreasing

## References

[b1] IUCN Red List Categories, IUCN Species Survival Commission, The World Conservation Union: http://www.iucnredlist.org (accessed: February 2013).

[b2] Abensperg-Traun M. CITES, sustainable use of wild species and incentive-driven conservation in developing countries, with an emphasis on southern Africa. Biol. Conserv. 142, 948–963 (2009).

[b3] Messer K. D. Protecting endangered species: When are shoot-on-sight policies the only viable option to stop poaching? Ecol. Econ. 69, 2334–2340 (2010).

[b4] Tobe S. S. & Linacre A. A new assay for identifying endangered species in Traditional East Asian Medicine. Forensic. Sci. Int. 3, e232–e233 (2011).

[b5] But P. P., Lung L. C. & Tam Y. K. Ethnopharmacology of rhinoceros horn. I: Antipyretic effects of rhinoceros horn and other animal horns. J. Ethnopharmacol. 30, 157–168 (1990). 2255207 10.1016/0378-8741(90)90005-e

[b6] Feng Z. *et al.* Molecular characteristics of Tibetan antelope (*Pantholops hodgsonii*) mitochondrial DNA control region and phylogenetic inferences with related species. Small Ruminant Res. 75, 236–242 (2008).

[b7] Peppin L., McEwing R., Carvalho G. R. & Ogden R. A DNA-based approach for the forensic identification of Asiatic black bear (*Ursus thibetanus*) in a traditional Asian medicine. J Forensic Sci. 53, 1358–1362 (2008). 18752556 10.1111/j.1556-4029.2008.00857.x

[b8] Coghlan M. L. *et al.* Deep sequencing of plant and animal DNA contained within traditional Chinese medicines reveals legality issues and health safety concerns. PLoS Genet. 8, e1002657 (2012). 22511890 10.1371/journal.pgen.1002657 PMC3325194

[b9] Schindel D. E. & Miller S. E. DNA barcoding a useful tool for taxonomists. Nature 435, 17 (2005). 15874991 10.1038/435017b

[b10] Blaxter M. Molecular systematics: counting angels with DNA. Nature 421, 122–124 (2003). 12520286 10.1038/421122a

[b11] Marshall E. Will DNA barcodes breathe life into classification? Science 307, 1037 (2005). 15718446 10.1126/science.307.5712.1037

[b12] Zhang Y., Shaw P., Sze C., Wang Z. & Tong Y. Molecular authentication of Chinese herbal materials. J. Food Drug Anal. 15, 1–9 (2007).

[b13] Feng Y. B. *et al.* Bear bile: dilemma of traditional medicinal use and animal protection. J. Ethnobiol. Ethnomed. 5, 1–9 (2009). 19138420 10.1186/1746-4269-5-2 PMC2630947

[b14] But P. P., Tam Y. K. & Lung C. Ethnopharmacology of rhinoceros horn II: antipyretic effects of prescriptions containing rhinoceros horn or water-buffalo horn. J. Ethnopharmacol. 33, 45–50 (1991). 1943172 10.1016/0378-8741(91)90159-b

[b15] Liu R., Wang M., Duan J. A., Guo J. M. & Tang Y. P. Purification and identification of three novel antioxidant peptides from *Cornu* *Bubali* (water buffalo horn). Peptides. 31, 786–793 (2010). 20206218 10.1016/j.peptides.2010.02.016

[b16] Tamura K., Dudley J., Nei M. & Kumars S. MEGA4: molecular evolutionary genetics analysis (MEGA) software version 4.0. Mol. Biol. Evol. 24, 1596–1599 (2007). 17488738 10.1093/molbev/msm092

[b17] Lahaye R. *et al.* DNA barcoding the floras of biodiversity hotspots. Proc. Natl. Acad. Sci. U.S.A. 105, 2923–2928 (2008). 18258745 10.1073/pnas.0709936105 PMC2268561

[b18] Meyer C. P. & Paulay G. DNA barcoding: error rates based on comprehensive sampling. PLoS Biol. 3, e422 (2005). 16336051 10.1371/journal.pbio.0030422 PMC1287506

[b19] Karikó K., Weissman D. & Welsh F. A. Inhibition of toll-like receptor and cytokine signaling--a unifying theme in ischemic tolerance. J. Cereb. Blood Flow Metab. 24, 1288–1304 (2004). 15545925 10.1097/01.WCB.0000145666.68576.71

[b20] Lastres-Becker I., Cartmell T. & Molina-Holgado F. Endotoxin preconditioning protects neurones from *in vitro* ischemia: role of endogenous IL-1beta and TNF-alpha. J. Neuroimmunol. 173, 108–116 (2006). 16439029 10.1016/j.jneuroim.2005.12.006

[b21] Brown J. W., Miller S. E. & Horak M. Studies on New Guinea moths. 2. Description of a new species of Xenothictis Meyrick (*Lepidoptera*: *Tortricidae*: *Archipini*). Proc. Entomol. Soc. Wash. 105, 1043–1050 (2003).

[b22] Chen H. *et al.* Toxicity of three phenolic compounds and their mixtures on the gram-positive bacteria *Bacillus subtilis* in the aquatic environment. Sci. Total Environ. 408, 1043–1049 (2010). 20006374 10.1016/j.scitotenv.2009.11.051

[b23] Zhao Y. L. *et al.* Study on the cold and hot properties of medicinal herbs by thermotropism in mice behavior. J. Ethnopharmacol. 133, 980–985 (2011). 20883763 10.1016/j.jep.2010.09.014

[b24] Yamasaki Y., Ikenaga T., Otsuki T., Nishikawa M. & Takakura Y. Induction of antigen-specific cytotoxic T lymphocytes by immunization with negatively charged soluble antigen through scavenger receptor-mediated delivery. Vaccine 25, 85–91 (2007). 16956699 10.1016/j.vaccine.2006.07.017

[b25] Suda T. *et al.* The route of immunization with adenoviral vaccine influences the recruitment of cytotoxic T lymphocytes in the lung that provide potent protection from influenza A virus. Antiviral. Res. 91, 252–258 (2011). 21722671 10.1016/j.antiviral.2011.06.008

[b26] O'Donnell M. J. & Quinlan M. C. Anti-diuresis in the blood-feeding insect Rhodnius prolixus Stål: antagonistic actions of cAMP and cGMP and the role of organic acid transport. J. Insect Physiol. 44, 561–568 (1998). 12769938 10.1016/s0022-1910(98)00047-x

[b27] Jang E. K., Davidson M. M., Crankshaw D. & Haslam R. J. Synergistic inhibitory effects of atriopeptin II and isoproterenol on contraction of rat aortic smooth muscle: roles of cGMP and cAMP. Eur. J. Pharmacol. 250, 477–481 (1993). 8112409 10.1016/0014-2999(93)90038-j

[b28] Luo J. Y. *et al.* Substitutes for endangered medicinal animal horns and shells exposed by antithrombotic and anticoagulation effects. J. Ethopharmacol. 136, 210–216 (2011). 10.1016/j.jep.2011.04.053 21549826

[b29] Guha S., Goyal S. P. & Kashyap V. K. Molecular phylogeny of musk deer: a genomic view with mitochondrial 16S rRNA and cytochrome *b* gene. Mol. Phylogenet. Evol. 42, 585–597 (2007). 17158073 10.1016/j.ympev.2006.06.020

[b30] Arif I. A., Bakir M. A. & Khan H. A. Inferring the phylogeny of *bovidae* using mitochondrial DNA sequences: resolving power of individual genes relative to complete genomes. Evol. Bioinform. 8, 139–150 (2012). 10.4137/EBO.S8897 PMC3290115 22399841

[b31] Hassanin A. & Douzery E. J. Molecular and morphological phylogenies of ruminantia and the alternative position of the *moschidae*. Syst. Biol. 52, 206–228 (2003). 12746147 10.1080/10635150390192726

[b32] Miller S. E. DNA barcoding and the renaissance of taxonomy. Proc. Natl. Acad. Sci. U.S.A. 104, 4775–4776 (2007). 17363473 10.1073/pnas.0700466104 PMC1829212

[b33] Bitanyi S. *et al.* Species identification of Tanzanian antelopes using DNA barcoding. Mol. Ecol. Resour. 11, 442–449 (2011). 21481202 10.1111/j.1755-0998.2011.02980.x

[b34] Hebert P. D., Penton E. H., Burns J. M., Janzen D. H. & Hallwachs W. Ten species in one: DNA barcoding reveals cryptic species in the neotropical skipper butterfly *Astraptes fulgerator*. Proc. Natl. Acad. Sci. U.S.A. 101, 14812–14817 (2004). 15465915 10.1073/pnas.0406166101 PMC522015

[b35] Magnacca K. N. & Brown M. J. DNA barcoding a regional fauna: Irish solitary bees. Mol. Ecol. Resour. 12, 990–998 (2012). 22931682 10.1111/1755-0998.12001

[b36] Reid B. N. *et al.* Comparing and combining distance-based and character-based approaches for barcoding turtles. Mol. Ecol. Resour. 211, 956–967 (2011). 21635698 10.1111/j.1755-0998.2011.03032.x

[b37] Francis C. M. *et al.* The role of DNA barcodes in understanding and conservation of mammal diversity in southeast Asia. PLoS One 5, e12575 (2010). 20838635 10.1371/journal.pone.0012575 PMC2933245

[b38] Lei T. *et al.* Antimicrobial resistance in *Escherichia coli* isolates from food animals, animal food products and companion animals in China. Vet. Microbiol. 146, 85–89 (2010). 20605690 10.1016/j.vetmic.2010.04.025

[b39] Suzuki H. & Yamamoto S. Campylobacter contamination in retail poultry meats and by-products in the world: a literature survey. J. Vet. Med. Sci. 71, 255–261 (2009). 19346690 10.1292/jvms.71.255

[b40] Alves R. R., Léo Neto N. A., Brooks S. E. & Albuquerque U. P. Commercialization of animal-derived remedies as complementary medicine in the semi-arid region of Northeastern Brazil. J. Ethnopharmacol. 124, 600–608 (2009). 19422902 10.1016/j.jep.2009.04.049

[b41] Patel V., Janssen M. & Sasso R. 88. P-15 Graft Substitute, Mechanism and Function. Spine J. 7, 43S (2007).

[b42] Gaasbeek R. D., Toonen H. G., van Heerwaarden R. J. & Buma P. Mechanism of bone incorporation of beta-TCP bone substitute in open wedge tibial osteotomy in patients. Biomaterials 26, 6713–6719 (2005). 15950278 10.1016/j.biomaterials.2005.04.056

[b43] Morimoto N. *et al.* The utilization of animal product-free media and autologous serum in an autologous dermal substitute culture. J. Surg. Res. 171, 339–346 (2011). 20189600 10.1016/j.jss.2009.11.724

[b44] Feng Y. B. *et al.* Bear bile: dilemma of traditional medicinal use and animal protection. J. Ethnobiol. Ethnomed. 5, 1–9 (2009). 19138420 10.1186/1746-4269-5-2 PMC2630947

[b45] Possingham H. P. *et al.* Limits to the use of threatened species lists. Trends Ecol. Evol. 17, 503–507 (2002).

[b46] United Nations Environment Programme and World Conservation Monitoring Centre. Checklist of CITES species: http://www.cites.org/eng/resources/pub/checklist11/index.html (accessed: February 2013).

[b47] Alves R. R. N. & Alves H. N. The faunal drugstore: animal-based remedies used in traditional medicines in Latin America. J. Ethnobiol. Ethnomed. 7, 1–43 (2011). 21385357 10.1186/1746-4269-7-9 PMC3060860

[b48] Alves R. R. N., Souto W. M. S. & Barboza R. R. D. Primates in traditional folk medicine: a world overview. Mammal Rev. 40, 155–180 (2011).

[b49] Alves R. R. N., Barboza R. R. D. & Souto W. M. S. A Global overview of canids used in traditional medicines. Biodivers. Conserv. 19, 1513–1522 (2010).

[b50] Alves R. R. N., Vieira W. L. S. & Santana G. G. Reptiles used in traditional folk medicine: conservation implications. Biodivers. Conserv. 17, 2037–2049 (2008).

[b51] Alves R. R. N. & Rosa I. L. Trade of Animals Used in Brazilian Traditional Medicine: Trends and Implications for Conservation. Human Ecol. 38, 691–704 (2010).

[b52] Whiting M. J., Williams V. L. & Hibbitts T. J. Animals traded for traditional medicine at the Faraday market in South Africa: species diversity and conservation implications. J. Zool. 284, 84–96 (2011).

[b53] Oliveira E. S., Torres D. F., Brooks S. E. & Alves R. R. N. The medicinal animal markets in the metropolitan region of Natal City, northeastern Brazil. J. Ethnopharmacol. 130, 54–60 (2010). 20460145 10.1016/j.jep.2010.04.010

[b54] Alves R. R. N. & Pereira Filho G. A. Commercialization and use of snakes in North and Northeastern Brazil: implications for conservation and management. Biodivers. Conserv. 16, 969–985 (2007).

[b55] Librado P. & Rozas J. DnaSP v5: a software for comprehensive analysis of DNA polymorphism data. Bioinformatics 25, 1451–1452 (2009). 19346325 10.1093/bioinformatics/btp187

[b56] Nei M. & Kumar S. Molecular Evolution and Phylogenetics. (Oxford University Press, 2000).

[b57] Ross H. A., Murugan S. & Li W. L. S. Testing the reliability of genetic methods of species identification via simulation. Syst. Biol. 57, 216–230 (2008). 18398767 10.1080/10635150802032990

[b58] Bruce S. J. *et al.* Investigation of human blood plasma sample preparation for performing metabolomics using ultrahigh performance liquid chromatography/mass spectrometry. Anal. Chem. 81, 3285–3296 (2009). 19323527 10.1021/ac8024569

[b59] Kong W. J. Spectrum-effect relationships between ultra performance liquid chromatography fingerprints and anti-bacterial activities of *Rhizoma coptidis*. Anal. Chim. Acta 634, 279–285 (2009). 19185133 10.1016/j.aca.2009.01.005

