# Supplementary material for: A strategy for trade monitoring and substitution of the organs of threatened animals
Source: Sci Rep. 2013 Oct 31;3:3108. doi: 10.1038/srep03108 (PMC3813934; doi:10.1038/srep03108)

**A strategy for trade monitoring and substitution of the organs of threatened animals**

**Jiao-yang Luo^1^, Dan Yan^1^, Jing-yuan Song^2^, Da Zhang^1^, Xiao-yan Xing^1^, Yu-mei Han^1^, Mei-hua Yang^2^, Xiao-ping Dong^3^, Cheng Peng^3^, Shi-lin Chen^2^, Xiao-he Xiao^1^**

**^1^** China Military Institute of Chinese Medicine, Integrative Medical Center of the 302 Military Hospital, Beijing 100039, P.R. China; ^2^ Institute of Medicinal Plant Development, Chinese Academy of Medical Sciences and Peking Union Medical College, Beijing 100094, P.R. China; **^3^** College of Pharmacy, Chengdu University of Chinese Traditional Medicine, Chengdu 610075, P.R. China

**Table S1.** GenBank accessions of 241 closely related species used to obtain ADDs; the COI sequences were cropped from the complete coding sequences and analyzed.

| GenBank accessions of close-related species of MAHS | | | | |
| --- | --- | --- | --- | --- |
| AB108504 | AB116261 | AB245426 | AB245427 | AF33441 |
| AF547270 | AY126697 | AY225986 | AY239042 | AY488491 |
| AY526085 | AY676855 | AY676856 | AY676857 | AY676858 |
| AY676859 | AY676860 | AY676861 | AY676862 | AY676863 |
| AY676864 | AY676865 | AY676866 | AY676867 | AY676868 |
| AY676869 | AY676870 | AY676871 | AY676872 | AY676873 |
| AY684273 | AY702618 | AY858379 | DQ124371 | DQ124372 |
| DQ124375 | DQ124376 | DQ124377 | DQ124378 | DQ124379 |
| DQ124380 | DQ124381 | DQ124382 | DQ124383 | DQ124384 |
| DQ124385 | DQ124386 | DQ124387 | DQ124388 | DQ124389 |
| DQ124390 | DQ124391 | DQ124392 | DQ124393 | DQ124394 |
| DQ124395 | DQ124396 | DQ124397 | DQ124398 | DQ124399 |
| DQ124400 | DQ124401 | DQ124402 | DQ124403 | DQ124404 |
| DQ124405 | DQ124406 | DQ124407 | DQ124408 | DQ124409 |
| DQ124410 | DQ124411 | DQ124412 | DQ124413 | DQ124414 |
| DQ124415 | DQ124416 | DQ124417 | DQ124418 | DQ347622 |
| DQ347626 | DQ989636 | EF035447 | EF050073 | EF494177 |
| EF494178 | EF494179 | EU177815 | EU177816 | EU177817 |
| EU177818 | EU177819 | EU177820 | EU177821 | EU177822 |
| EU177823 | EU177824 | EU177825 | EU177826 | EU177827 |
| EU177828 | EU177829 | EU177830 | EU177831 | EU177832 |
| EU177833 | EU177834 | EU177835 | EU177836 | EU177837 |
| EU177838 | EU177839 | EU177840 | EU177841 | EU177842 |
| EU177843 | EU177844 | EU177845 | EU177846 | EU177847 |
| EU177848 | EU177849 | EU177850 | EU177851 | EU177852 |
| EU177853 | EU177854 | EU177855 | EU177856 | EU177857 |
| EU177858 | EU177859 | EU177860 | EU177861 | EU177862 |
| EU177863 | EU177864 | EU177865 | EU177866 | EU177867 |
| EU177868 | EU177869 | EU177870 | EU177871 | EU315254 |
| EU708434 | EU809939 | FJ171914 | FJ171915 | FJ469673 |
| FJ763736 | FJ890514 | FJ971080 | FJ971081 | FJ971082 |
| FJ971083 | FJ971084 | FJ971085 | FJ971086 | FJ971087 |
| FJ971088 | FJ997262 | FJ185309 | FJ461529 | FJ461530 |
| FJ461531 | GQ129207 | GQ856370 | GQ129208 | GU068049 |
| GU229278 | GU229279 | GU229280 | GU229281 | GU457433 |
| GU457434 | GU457435 | GU563918 | GU985279 | HM045017 |
| HM045018 | JN190944 | JN190945 | JN190946 | JN315436 |
| JN315437 | JN315438 | JN315439 | JN315440 | JN315441 |
| JN315442 | JN315443 | JN417004 | NC_001941 | NC_002780 |
| NC_004027 | NC_004069 | NC_004563 | NC_004577 | NC_005044 |
| NC_005971 | NC_006082 | NC_006132 | NC_006295 | NC_006380 |
| NC_006853 | NC_006973 | NC_006993 | NC_007179 | NC_007441 |
| NC_007703 | NC_008414 | NC_008462 | NC_008491 | NC_008749 |
| NC_009330 | NC_009509 | NC_009510 | NC_010640 | NC_010970 |
| NC_010973 | NC_011819 | NC_011821 | NC_012054 | NC_012096 |
| NC_012098 | NC_012346 | NC_012414 | NC_012706 | NC_012833 |
| NC_013069 | NC_013751 | NC_013834 | NC_013836 | NC_013840 |
| NC_013841 | NC_013996 | NC_014044 | NC_014054 | NC_014102 |
| X61145 |  |  |  |  |

**Table S2.** Endotoxin contents in rabbit sera at 1.5 h as determined using a dynamic nephelometric method.

|  | Dilution multiple | Extra endotoxin concentration (EU/mL) | Recovery  (%) | Reaction time (s) | Coefficient of variability (%) | Measured Value (EU/mL) | Reference value (EU/mL) |
| --- | --- | --- | --- | --- | --- | --- | --- |
| velvet antler | 40 | 0 | － | 3115 | 0.00 | 0.0214 | 0.856 |
|  | 40 | 0.2 | 88.15 | 1258 | 0.84 | 0.1947 | － |
| antler | 40 | 0 | － | ＞3600 | 0.00 | ＜0.013 | ＜0.520 |
|  | 40 | 0.2 | 100.25 | 1271 | 1.53 | 0.2266 | － |
| saiga antelope | 40 | 0 | － | ＞3600 | 0.00 | ＜0.013 | ＜0.520 |
|  | 40 | 0.2 | 93.85 | 1253 | 1.24 | 0.2287 | － |
| domestic goat | 40 | 0 | － | ＞3600 | 0.00 | ＜0.013 | ＜0.520 |
|  | 40 | 0.2 | 136.1 | 1248 | 3.51 | 0.2840 | － |
| negative control | 40 | 0 | － | ＞3600 | 0.00 | ＜0.013 | ＜0.520 |
|  | 40 | 0.2 | 130.25 | 1221 | 2.58 | 0.2625 | － |

**Table S3.** Damage to spleen lymphocytes (cultured *in vitro*) under the influence of ADDs.

| **Concentration**  **(mg/mL)** | **The damage extent to spleen lymphocytes (cultured *in vitro*) influenced by MAHS (%)** | | | | | | |
| --- | --- | --- | --- | --- | --- | --- | --- |
|  | velvet antlter | antler | saiga antelope | domestic goat | Asian water buffalo | Chinese pangolin | Chinese softshell turtle |
| 100 | 35.83 | 47.38 | 91.42 | 59.84 | 87.95 | 88.34 | 85.27 |
| 80 | 0 | 0 | 70.86 | 23.57 | 47.42 | 52.37 | 41.26 |
| 60 | 0 | 0 | 38.52 | 0 | 21.85 | 33.47 | 26.39 |
| 40 | 0 | 0 | 0 | 0 | 0 | 0 | 0 |
| 20 | 0 | 0 | 0 | 0 | 0 | 0 | 0 |
| 10 | 0 | 0 | 0 | 0 | 0 | 0 | 0 |
| 5 | 0 | 0 | 0 | 0 | 0 | 0 | 0 |
| 2.5 | 0 | 0 | 0 | 0 | 0 | 0 | 0 |
| 1.25 | 0 | 0 | 0 | 0 | 0 | 0 | 0 |
| 0.625 | 0 | 0 | 0 | 0 | 0 | 0 | 0 |
| 0 | 0 | 0 | 0 | 0 | 0 | 0 | 0 |

**Table S4. A review of the status of threatened animals and their substitutes (the substitutes recommended by this study are included).**

| **Scientific name** | **Common name** | **IUCN Red List** | **CITES legislation** | **Pop. trend** | **Alternatives and scope** |
| --- | --- | --- | --- | --- | --- |
| **Cetacean** | | | | | |
| *Balaenoptera borealis* | Sei whale | [Endangered](http://en.wikipedia.org/wiki/Endangered) | Appendix I | Unknown | **×** |
| *Balaenoptera musculus* | Blue whale | Endangered | Appendix I | Increasing | **×** |
| *Balaenoptera physalus* | Fin whale | Endangered | Appendix I | Unknown | **×** |
| [*Physeter macrocephalus*](http://www.iucnredlist.org/details/41755/0) | Sperm whale | Vulnerable | Appendix I | Unknown | **√** Sperm oil  ⊙Jojoba oil  ◇Various lubricant applications, wetting agents and extreme pressure additives  ⊙Deep water fish species  *Hoplostethus atlanticus*  *Allocyttus* sp.  *Pseudocyttus maculatus*  ◇Liquid waxes, used in the cosmetic and high-grade lubricant fields |
| *Balaenoptera bonaerensis* | Antarctic minke whale | DD | Appendix I | Unknown | **×** |
| *Balaenoptera edeni* | Bryde's whales | DD | Appendix I | Unknown | **×** |
| *Balaenoptera omurai* | Omura’s whale | DD | Appendix I | Unknown | **×** |
| [**Carnivora**](app:ds:Carnivora) |  |  |  |  |  |
| *Panthera tigris* | Tiger | Endangered | Appendix I | Decreasing | **√**[Tiger-bone](app:ds:tiger-bone)(medical use)  ⊙*Myospalax baileyi*  ◇Curerheumatic diseases |
| *Ursus thibetanus* | Himalayan black bear | Vulnerable | Appendix I | Decreasing | **√**Bear bile(medical use)  ⊙Pig bile  ◇Anti-inflammatory,  anti-convulsion and analgesic effects.  ⊙Rabbit bile  ◇Positive inotropic action, sedation, anti-tussive action, and anti-histaminic action.  ⊙Coptis  △Clear heat, detoxify, purify liver, improve eyesight et al. |
| **Proboscidea** | | | | | |
| *Elephas maximus* | Asian elephant | Endangered | Appendix I | Decreasing | √[Ivory](app:ds:ivory)  ⊙Mammoth ivory, cow bone, camel bone  ◇Ivory carving |
| **Pholidota** | | | | | |
| *Manis pentadactyla* | Chinese pangolin | Endangered | Appendix II | Decreasing | √ pangolin scale(medicinal use)  ⊙turtle shell  △Immunoregulatory activity |
| **Perissodactyla** | | | | | |
| *Diceros bicornis* | Black rhinoceros | Critically endangered | Appendix I | Increasing | **√**Rhinoceros horn(medical use)  ⊙water buffalo horn  ◇Antipyretic effects |
| *Dicerorhinus sumatrensis* | Sumatran rhinoceros | Critically endangered | Appendix I | Decreasing |
| *Rhinoceros sondaicus* | Javan rhinoceros | Critically endangered | Appendix I | Unknown |
| *Rhinoceros unicornis* | Indian rhinoceros | Vulnerable | Appendix I | Increasing |
| *Ceratotherium simum* | White rhinoceros | Near threatened | Appendix I,II | Increasing |
| **Artiodactyla** | | | | | |
| *Elaphurus davidianus* | Père David's deer | Extinct in the wild | NL | Increasing | **×** |
| *Bos sauveli* | Kouprey | Critically endangered | Appendix I | Unknown | **×** |
| *Bubalus mindorensis* | Tamaraw | Critically endangered | Appendix I | Decreasing | **×** |
| *Nanger dama* | Dama gazelle | Critically endangered | Appendix I | Decreasing | **×** |
| *Saiga tatarica* | Mongolian saiga | Critically endangered | Appendix II | Decreasing | **√**Antelope's horn( medical use)  ⊙goat horn  ◇Immunological activity, sedation, analgesia  △ Antipyretic activity |
| *Pseudoryx nghetinhensis* | Saola | Critically endangered | Appendix I | Decreasing | **×** |
| *Axis porcinus* | Hog deer | Endangered | Appendix I | Decreasing | **×** |
| *Axis calamianensis* | Calamian hog deer | Endangered | Appendix I | Decreasing | **×** |
| *Gazella leptoceros* | Slender-horned gazelle | Endangered | Appendix I | Decreasing | **×** |
| *Bubalus depressicornis* | Lowland anoa | Endangered | Appendix I | Decreasing | **×** |
| *Hippocamelus bisulcus* | Chilean guemal | Endangered | NL | Decreasing | **×** |
| *Bosjavanicus* | Banteng | Endangered | NL | Decreasing | **×** |
| *Pantholops hodgsonii* | Chiru | Endangered | Appendix I | Decreasing | **×** |
| *Capra falconeri* | Markhor | Endangered | Appendix I | Decreasing | **×** |
| *Bubalus arnee* | Indian water buffalo | Endangered | Appendix III | Decreasing | **×** |
| *Moschus berezovskii* | Forest musk deer | Endangered | Appendix I,II | Decreasing | **√**Musk(medical use) |
| *Moschus chrysogaster* | Alpine musk deer | Endangered | Appendix I,II | Decreasing | ⊙*Ondatra zibethica*  ◇Anti-inflammation and analgesion97 |
| *Moschus moschiferus* | Siberian musk deer | Vulnerable | AppendixI,II | Decreasing |
| *Bos mutus* | Wild yak | Vulnerable | Appendix I | Decreasing | **√**Yak horn(medical use)  ⊙*Bubalus bubalis*  △Anticogulate activity |
| *Bison bonasus* | European bison | Vulnerable | NL | Increasing | **×** |
| *Oryx leucoryx* | Arabian oryx | Vulnerable | Appendix I | Stable | **×** |
| [*Eudorcas thomsonii*](http://www.iucnredlist.org/details/8982/0) | Thomson's gazelles | Near threatened | NL | Decreasing | **×** |
| *Procapra gutturosa* | Mongolian gazelle | Least concern | NL | Unknown | **×** |
| *Dama dama* | Fallow deer | Least concern | Appendix I | Unknown | **×** |
| *Cervus nippon* | Sika deer | Least concern | NL | Increasing | **√**[Pilos](app:ds:pilos) [deer](app:ds:deer) [horn](app:ds:horn)(medical use)  ⊙Calcinated antler cancellous bone  ◇Source of bone graft material |
| *Cervus elaphus hanglu* | Red deer | Least concern | Appendix I | Increasing | **×** |
| *Cervus elaphus bactrianus* | Red deer | Least concern | Appendix II | Increasing | **×** |
| *Cervus elaphus barbarous* | Red deer | Least concern | Appendix III | Increasing | **×** |
| **Reptile** | | | | | |
| *Eretmochelys imbricata* | Hawksbill turtle | Critically endangered | NL | Decreasing | **×** |
| *Pelochelys cantorii* | Frog-faced softshell turtle | Endangered | Appendix II | Needs updating | **×** |
| *Platysternon megacephalum* | Big-headed turtle | Endangered | Appendix II | Needs updating | **×** |
| *Caretta caretta* | Loggerhead | Endangered | NL | Needs updating | **×** |
| **Shark** | | | | | |
| *Sphyrna lewini* | Scalloped hammerhead shark | Endangered | Appendix III | Unknown | **×** |
| *Cetorhinus maximus* | Basking shark | Vulnerable | Appendix II | Decreasing | **×** |
| *Carcharodon carcharias* | Great white shark | Vulnerable | Appendix II | Unknown | **×** |

DD: data deficient; NL: not listed; √ medicinal parts or other applications; × no alternative use; ⊙substitutes; ◇alternative application scope; △ penitential recommendation by the present study.

**Figure S1. Distribution of some endangered and threatened species examined in this study.** (A) General distribution of the species in the wild; (B) detailed distribution of endangered and threatened species. (The information of the maps was derived from investments made in TM in China, and we hold the copyright of this figure.)


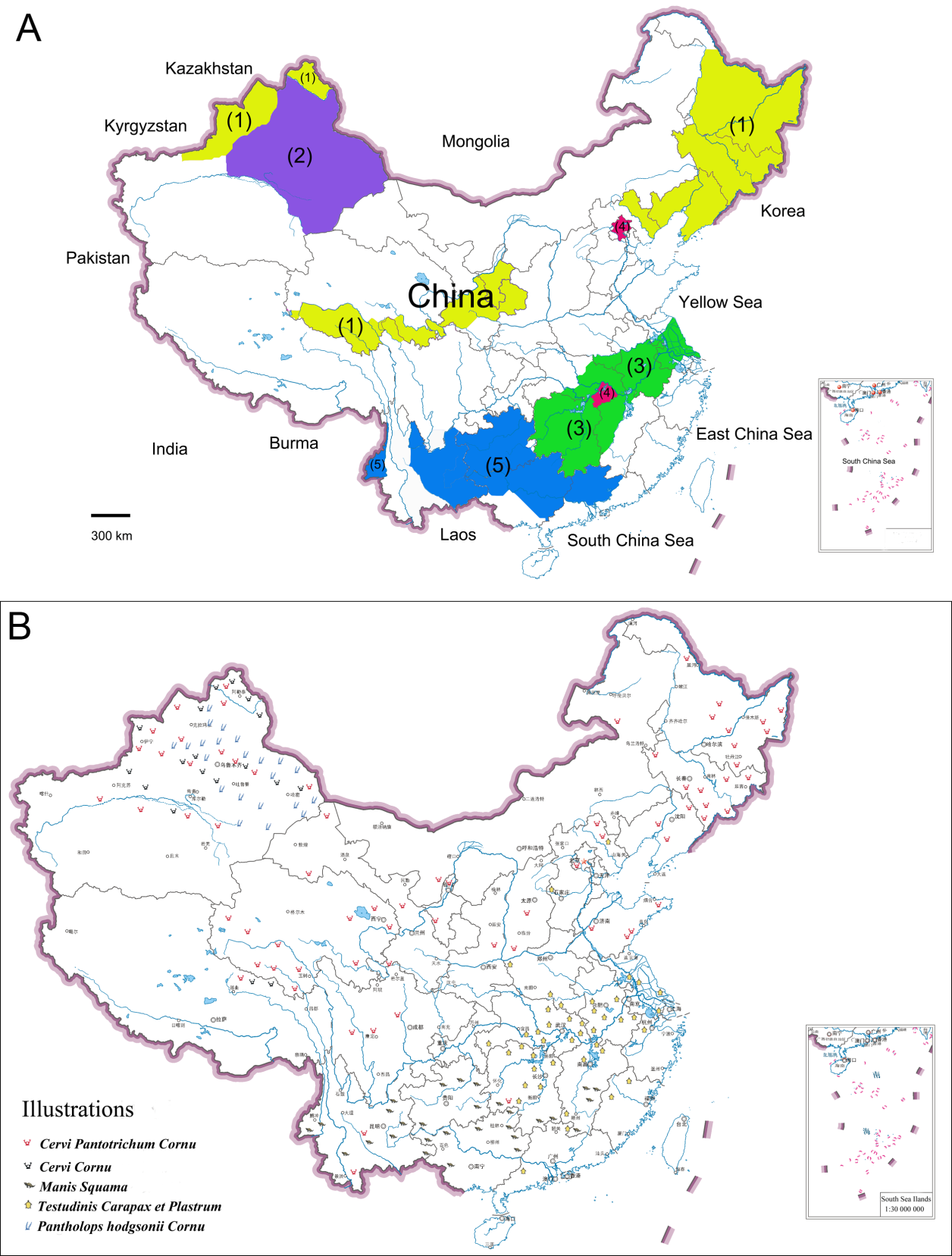


**Figure S2.** Neighbor-joining tree based on Kimura 2-parameter distances for 46 COI gene sequences.


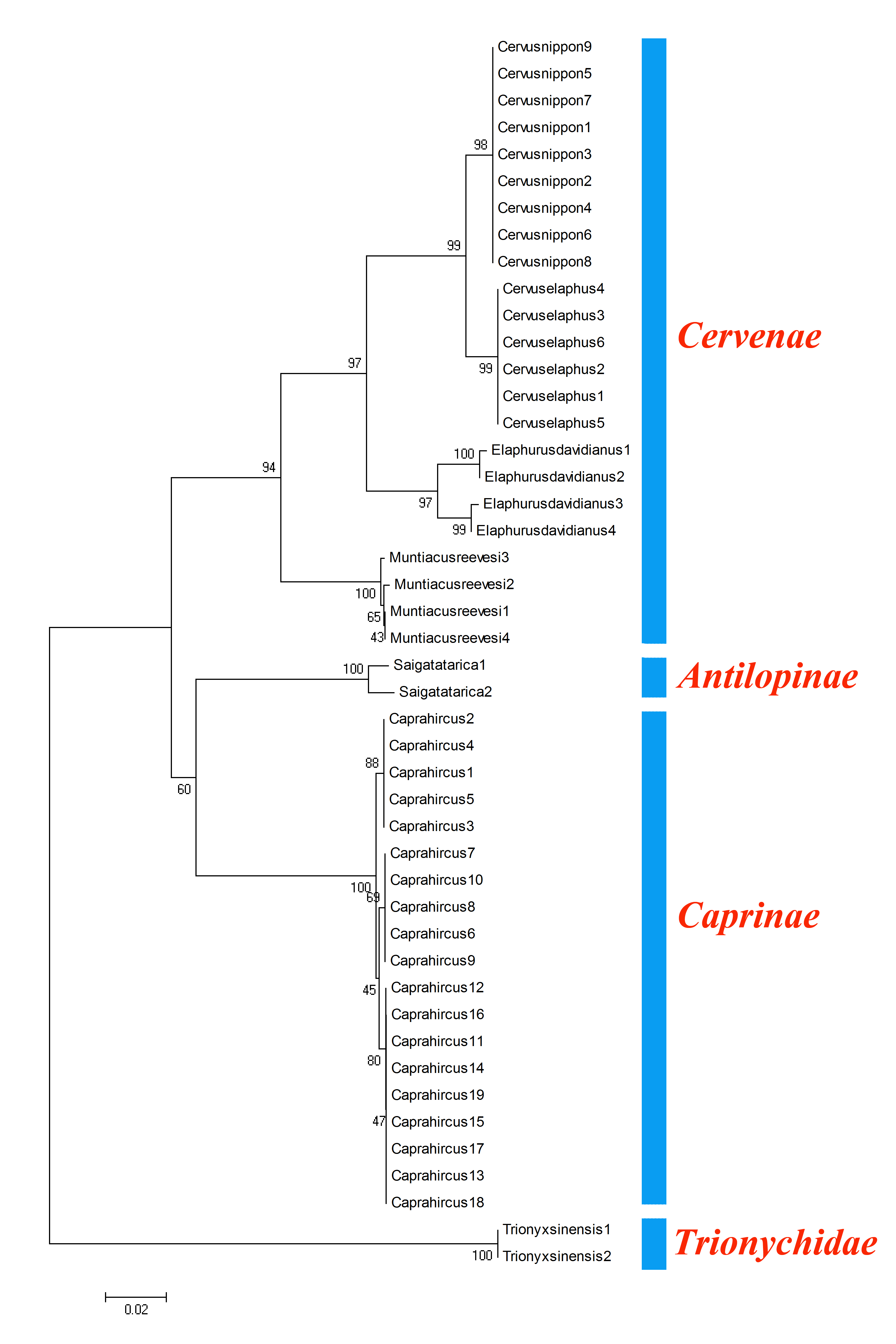


**Figure S3. Barcoding gap test.** The relative distribution of inter- and intraspecific variations.


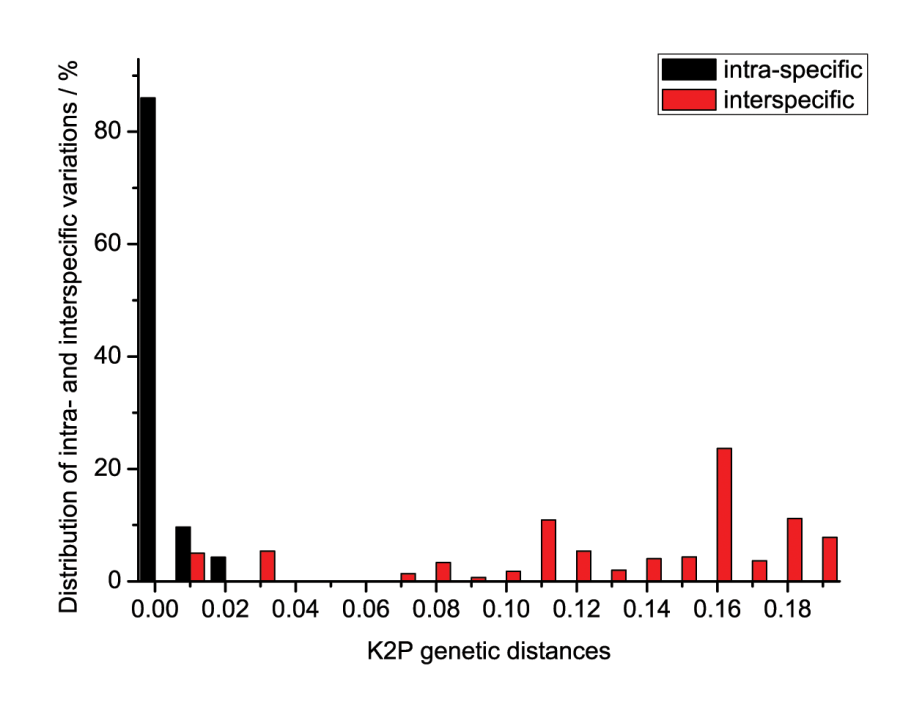


**Figure S4.** Antipyretic activities of ADDs. (A) Maximum changes in body temperature in the presence of various ADDs; (B) PGE2 level in rabbit serum; (C) PGE1 level in rabbit serum; (D) TNF-α level in rabbit serum; (E) d-values among the temperatures.


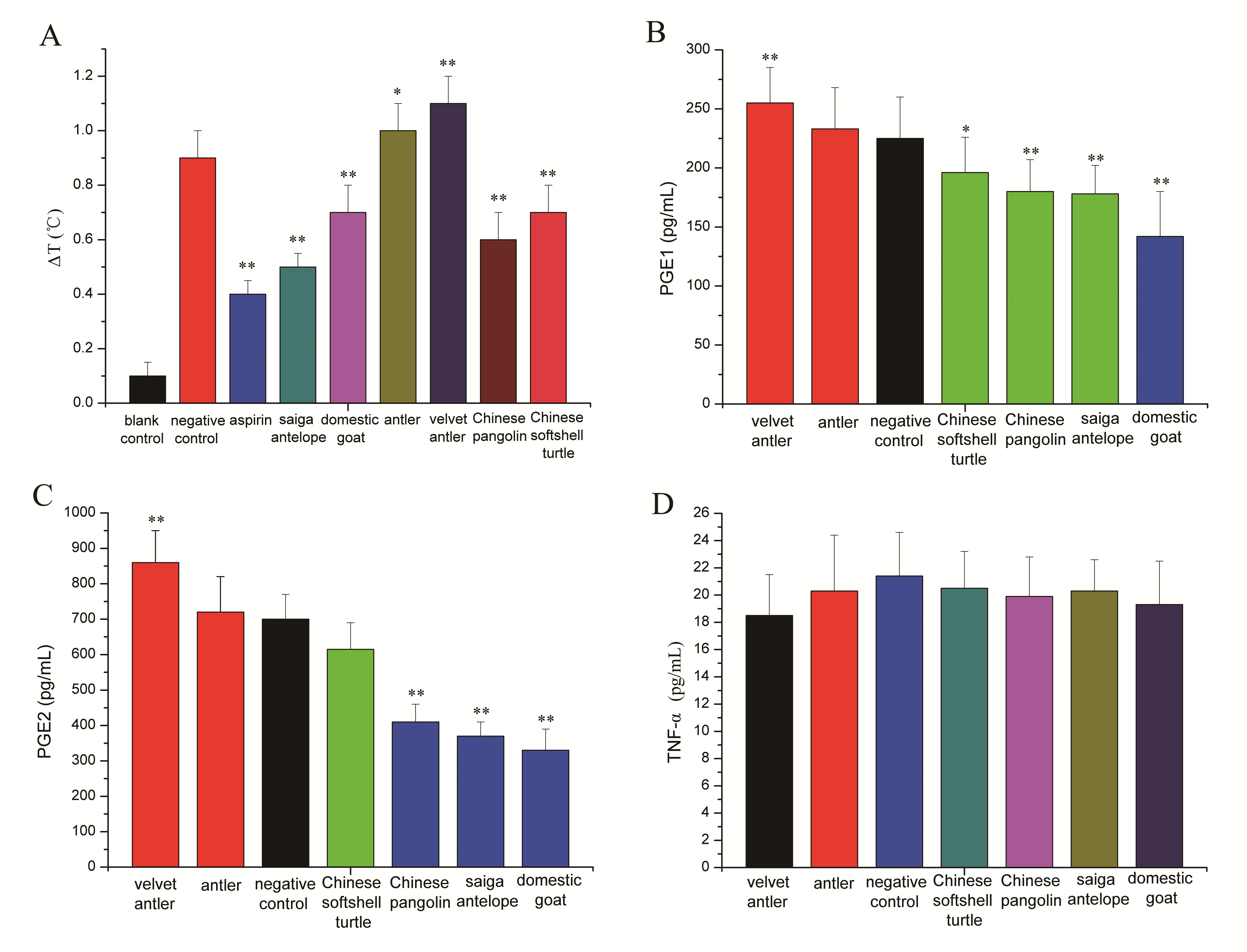


**Figure S5.** The effects of ADDs on liver microsomes. (A) HFP-t curves of the growth of liver microsomes in the presence of ADDs *in vitro*, within which (a)-(e) represent velvet antler group, antler group, blank control, domestic group and saiga antelope group, respectively; (B) total heat output of the heat-flow power under the action of various ADDs; (C) inhibition ratios of the ADDs on the growth of liver microsomes *in vitro*.


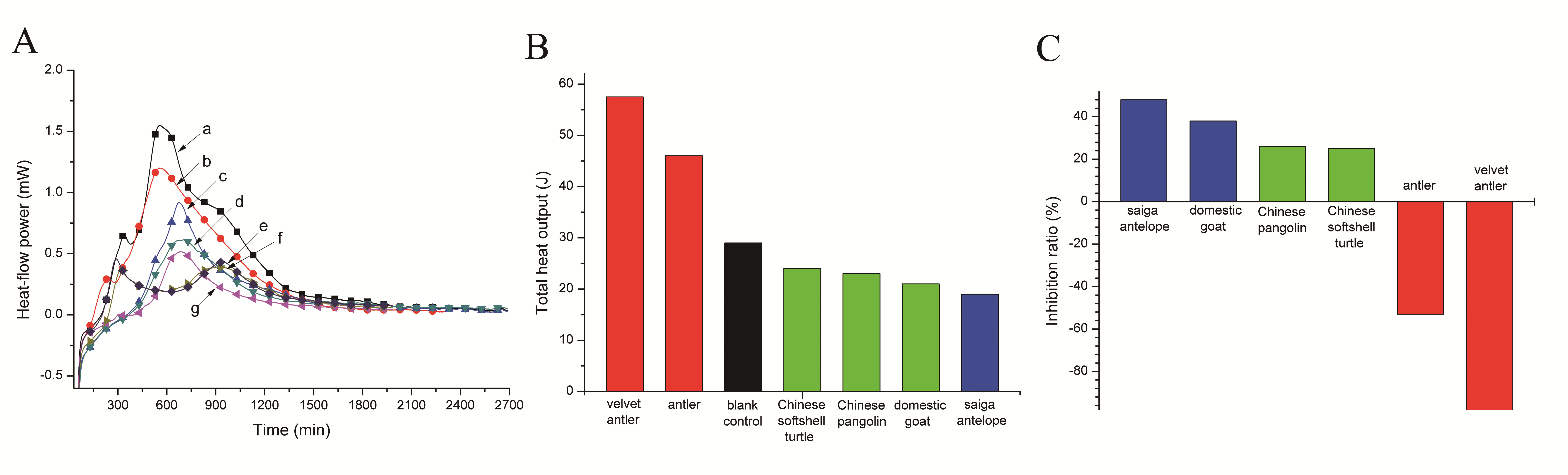


**Figure S6.** The effects of various ADDs on spleen lymphocytes. (A) Kunming mice; (B) cultured spleen lymphocytes; (C) microcalorimetry; (D) HFP-t curves of the growth and metabolism of spleen lymphocytes in the presence of ADDs, within which (a)-(h) represent velvet antler group, domestic goat group, antler group, blank group, Chinese pangolin group, saiga antelope group, Asian water buffalo group, and Chinese softshell turtle group, respectively; (E) parameters extracted from the HFP-t curves; (F) comparison of the values of the heat-flow power in the presence of various ADDs; (G) comparison of the values of the total heat output in the presence of various ADDs.


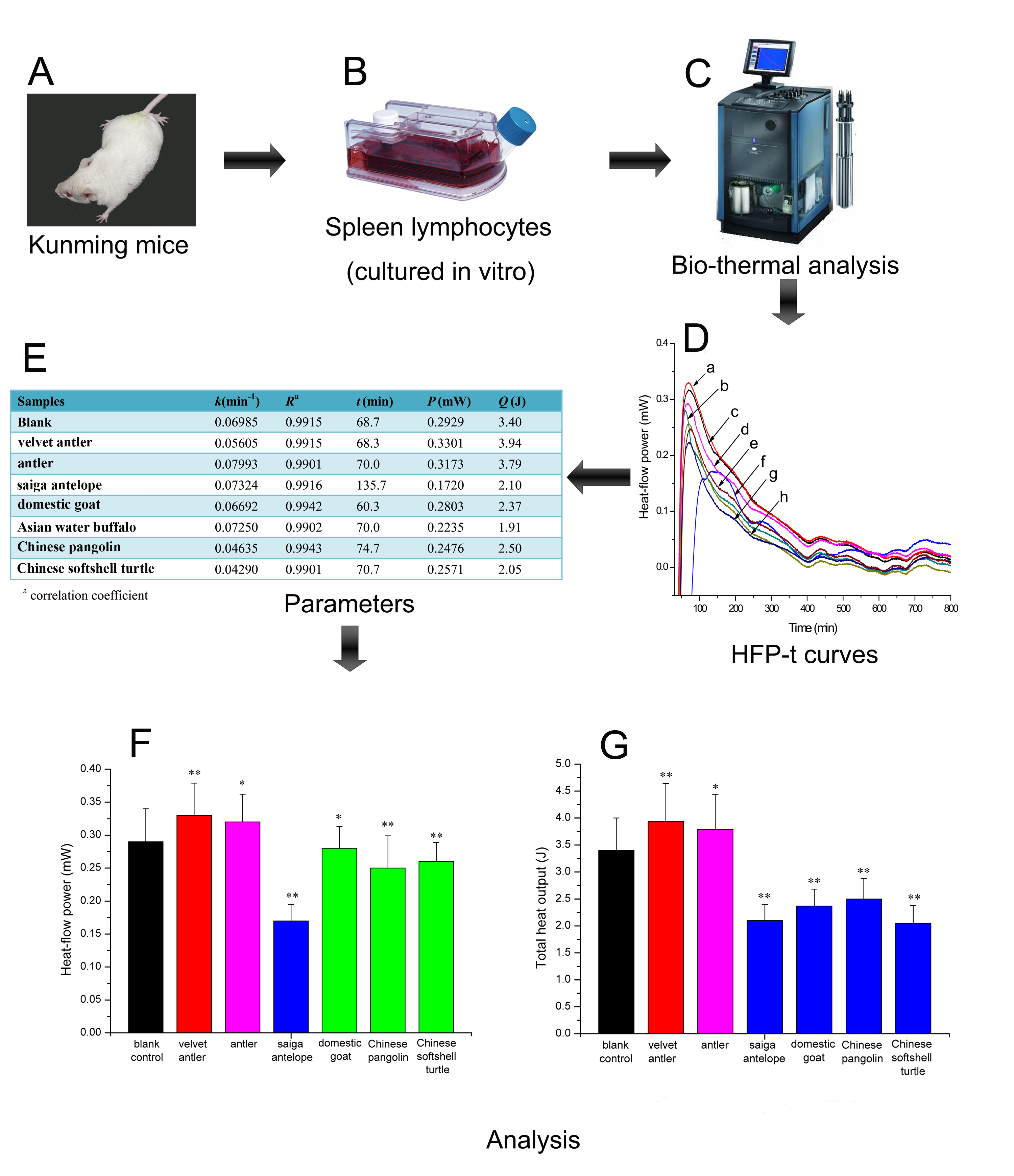


**Figure S7.** Investigation of the temperature gradients in the mouse thermotaxis study.


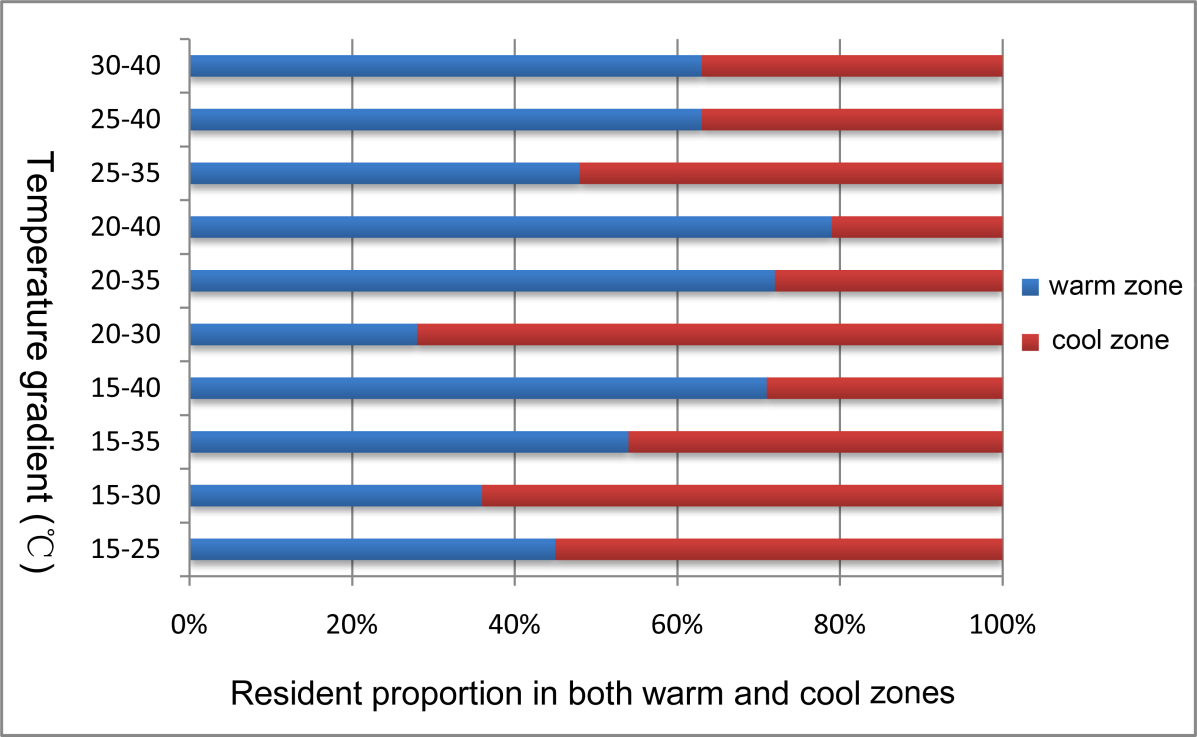


**Figure S8.** Investigation of the observation time in the mouse thermotaxis study.


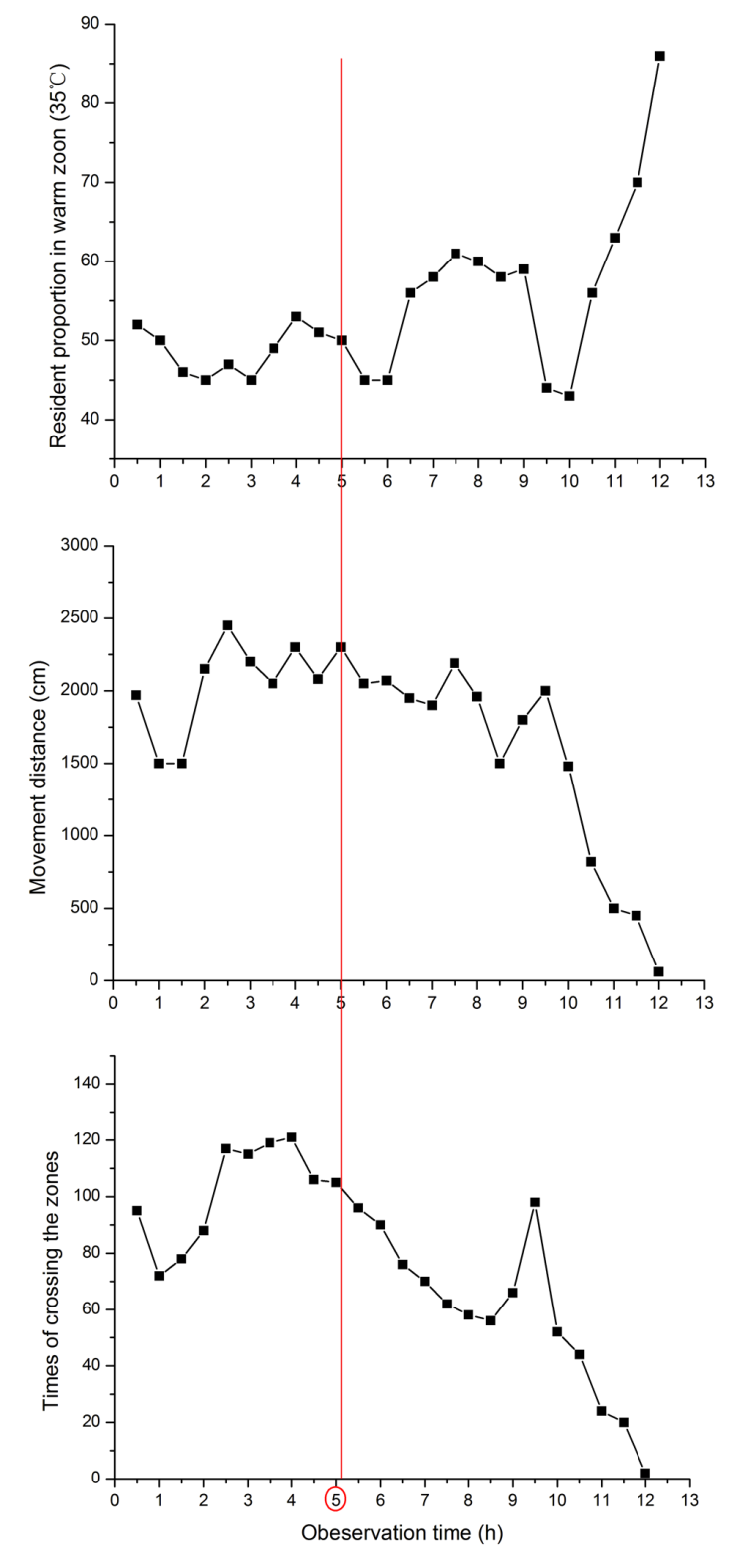


**Figure S9.** The effects of ADDs on mouse thermotaxis. (A) Temperature-controlled setup; (B) remote monitoring system; (C) data-processing system; (D): comparison of the proportion of animals in the warm zone in the presence of various ADDs; (E) comparison of the 30 min moving distances in the presence of various ADDs; (F) comparison of times taken to cross from one zone to another in the presence of various ADDs; (G) 5-HT levels in mouse sera; (H) cAMP levels in mouse sera; (I) cGMP levels in mouse sera; (J) cAMP/cGMP values.


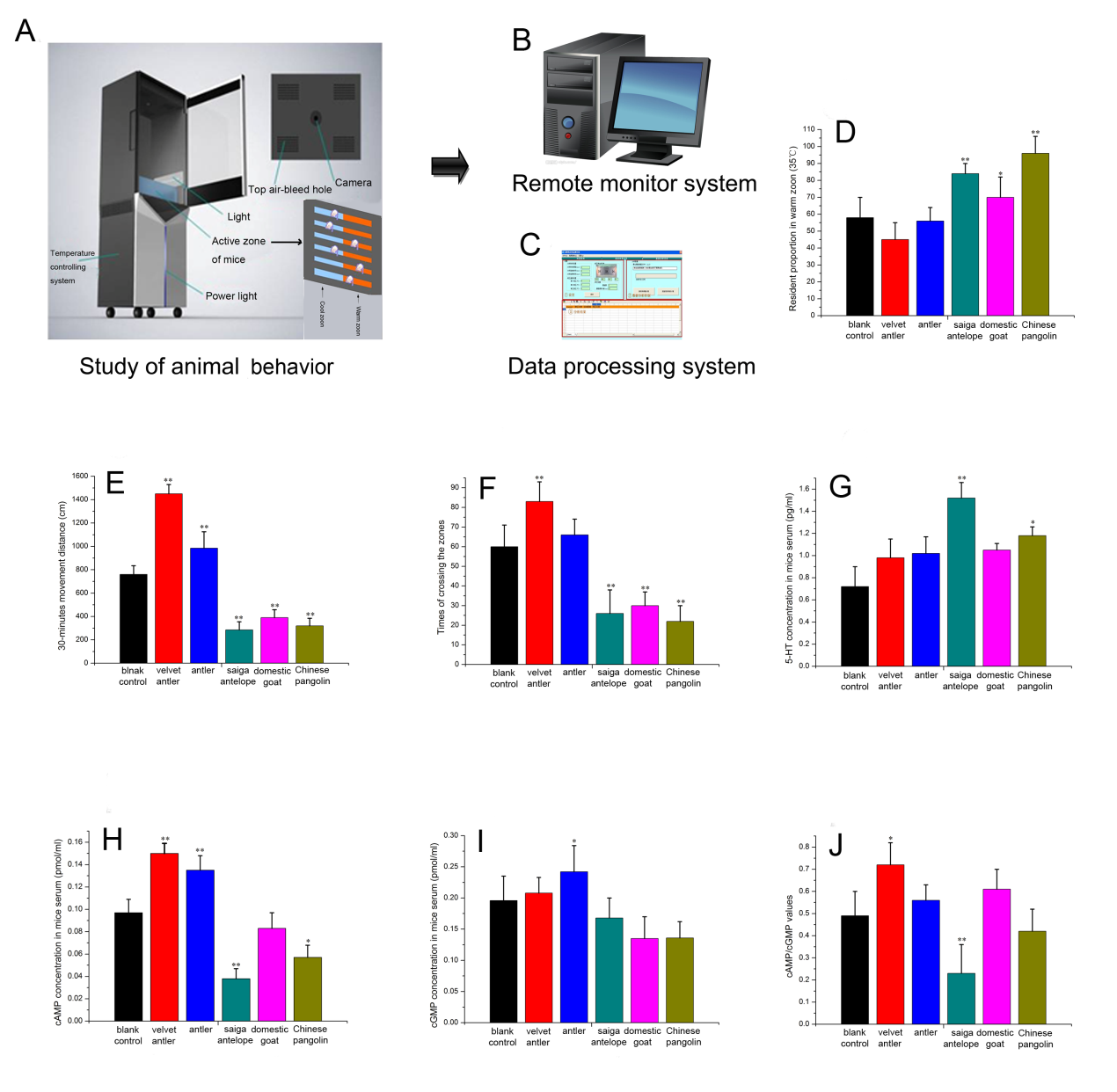


**Figure S10.** Determination of 5-HT, cAMP, and cGMP levels in the brain tissues of mice. (A) 5-HT level in the brain tissues of mice; (B) cAMP level in the brain tissues of mice; (C) cGMP level in the brain tissues of mice; (D) cAMP /cGMP values.


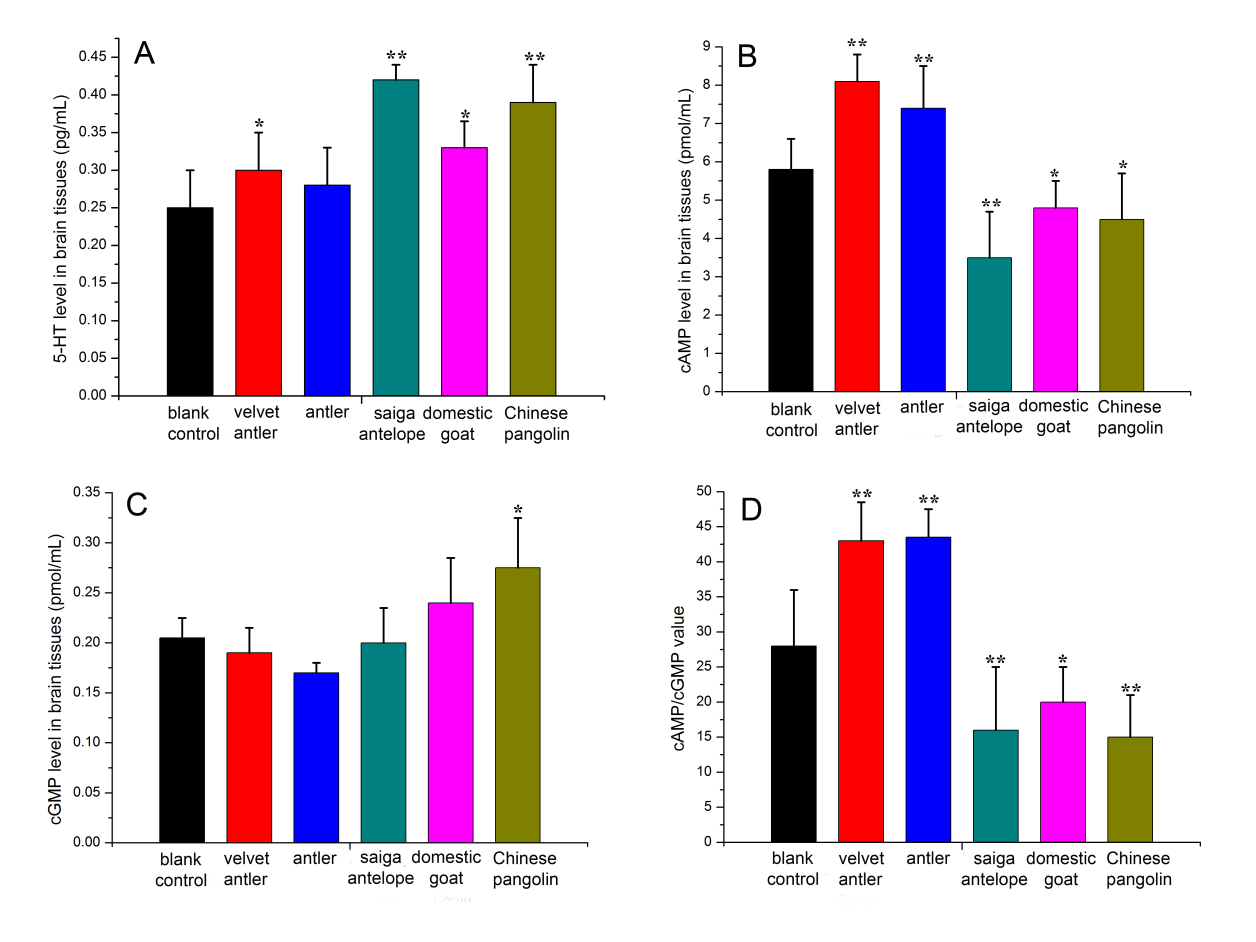

Supplement: Supplementary Information [file srep03108-s1.doc]
